# Linking cerebral hemodynamics and ocular microgravity-induced alterations through an in silico-in vivo head-down tilt framework

**DOI:** 10.1038/s41526-024-00366-8

**Published:** 2024-02-27

**Authors:** Matteo Fois, Ana Diaz-Artiles, Syeda Yasmin Zaman, Luca Ridolfi, Stefania Scarsoglio

**Affiliations:** 1 Department of Mechanical and Aerospace Engineering, Politecnico di Torino, Corso Duca degli Abruzzi 24, Turin, 10129 Italy; 2 Department of Aerospace Engineering, Texas A&M University, 3141 TAMU, College Station, TX 77843-3141 USA; 3 Department of Kinesiology and Sport Management, Texas A&M University, 2929 Research Pkwy, College Station, TX 77845 USA; 4 Department of Environmental, Land and Infrastructure Engineering, Politecnico di Torino, Corso Duca degli Abruzzi 24, Turin, 10129 Italy; 5 PolitoBioMed Lab, Politecnico di Torino, Corso Duca degli Abruzzi 24, Turin, 10129 Italy

**Keywords:** Eye manifestations, Preventive medicine, Experimental models of disease

## Abstract

Head-down tilt (HDT) has been widely proposed as a terrestrial analog of microgravity and used also to investigate the occurrence of spaceflight-associated neuro-ocular syndrome (SANS), which is currently considered one of the major health risks for human spaceflight. We propose here an in vivo validated numerical framework to simulate the acute ocular-cerebrovascular response to 6° HDT, to explore the etiology and pathophysiology of SANS. The model links cerebral and ocular posture-induced hemodynamics, simulating the response of the main cerebrovascular mechanisms, as well as the relationship between intracranial and intraocular pressure to HDT. Our results from short-term (10 min) 6° HDT show increased hemodynamic pulsatility in the proximal-to-distal/capillary-venous cerebral direction, a marked decrease (-43%) in ocular translaminar pressure, and an increase (+31%) in ocular perfusion pressure, suggesting a plausible explanation of the underlying mechanisms at the onset of ocular globe deformation and edema formation over longer time scales.

## Introduction

The future of space travel and exploration relies on our knowledge of human physiological adaptation to the space environment^1^ as well as on the support of technological advancement. Ensuring the health and survival of astronauts undergoing short- and long-term permanence in space is a crucial goal of bioastronautics^2,3^ and space medicine^4^. In particular, spaceflight-associated neuro-ocular syndrome (SANS) has been acknowledged by NASA as a major risk for human space travel^5^, requiring mitigation strategies in view of future crewed expeditions to Mars^6^. The syndrome includes a number of ophthalmic anomalies and neuro-ocular changes observed in astronauts returning from the International Space Station (ISS)^7^, and these changes may persist permanently after re-entry^8^.

At present, the exact etiology and pathophysiology of SANS still remain unknown. It is hypothesized that cephalic vascular and subarachnoid volume engorgement driven by headward fluid shift, mildly elevated intracranial pressure (ICP), and elevated cerebral blood volume experienced by astronauts in microgravity may play a role over long time scales^9–17^. For this reason, the human cardiovascular system (CVS) and its response/adaptation to weightlessness have been extensively investigated in association with SANS^11,18,19^. However, the understanding of the cerebrovascular circulation response to weightlessness and its relationship to SANS still remains unclear.

Over recent years, ground-based analogs of spaceflight have been adopted to reproduce the acute and long-term effects of microgravity on cardiovascular physiology^18,20–22^. Among these, 6° head-down tilt (HDT) has gathered wide popularity to simulate the fluid shift experienced in weightlessness on human circulation^23^. Remarkable efforts have been dedicated to investigating and identifying SANS risks, causes, and potential countermeasures by resorting to 6° HDT as a terrestrial analog of spaceflight^12,15,24–31^. Some of these studies focused on the relationship between the internal pressure of the eye globe, i.e., the intraocular pressure (IOP), the pressure in the retrobulbar space (ICP), and blood pressure at the eye level (especially arterial pressure) determining perfusion of the globe. However, despite previous investigations about cerebrovascular responsiveness during HDT^29,32–34^ and parabolic flight^35^, the link between cerebral hemodynamics (underlying ICP and eye arterial pressure) and the ocular apparatus is still poorly understood. The pressure difference between IOP and ICP called ocular translaminar pressure (TLP), is believed to play a relevant role in the development of SANS. In particular, reduced (or reversed) TLP may compromise the spherical shape of the eye^13–15,25^. In addition, elevated ocular perfusion pressure (OPP, defined as the difference between eye arterial pressure and IOP) has also been hypothesized as a potential risk for edema formation^31^. Further ground and in-flight in vivo investigations are necessary to confirm the exact role of TLP and OPP in the development of SANS. A number of IOP measurements have been taken during acute and long exposure to microgravity^36–40^. Unfortunately, given the harmful and difficult nature of invasive measurements for ICP^41,42^, in vivo reference values associated with posture variation (e.g., during 6° HDT) are rare in the literature^43,44^, while ICP data onboard space missions are still lacking to date (albeit some results have been reported during parabolic flight^13^).

In this context, mathematical modeling represents a powerful tool to investigate hidden or unclear physiological mechanisms of the CVS under a variety of “altered-gravity” conditions. Over recent years, mathematical modeling of the CVS has been used to study the cardiovascular responses to head-up tilt^45–47^, to centrifugation (with and without exercise)^48^, cardiovascular deconditioning following long-term spaceflight^49^, the impact of orthostatic stress and acute weightlessness on the cerebrovascular dynamics^50^, or the arterial-ocular interaction (IOP, OPP) during head-up and head-down tilt^31,51^. We aim to provide an in silico-in vivo framework to link the gravity-driven cerebrovascular and ocular mechanisms. This can lead to better elucidation of the ICP and IOP responses to altered-gravity, contributing to the understanding of the pathophysiology of SANS.

Therefore, in the present work, we present a multiscale model of the human circulation integrating our previous 1D-0D CVS framework^47,52^ with a lumped model of the ocular compartments^31,51^ and with a lumped-parameter model of the cerebrovascular circulation^53^. The lumped ocular model encompasses multiple compartments and depends on both arterial and venous pressures at the level of the eye, as well as on ICP. The lumped cerebrovascular model accounts for the mechanisms of cerebral autoregulation and CO2 reactivity, and it has been validated and previously used to investigate the effects of cardiac arrhythmias on cerebral hemodynamics^54–57^. More recently, the lumped cerebrovascular model has also been used to explore the gravity-induced cerebrovascular dynamics during simulated parabolic flight^58^.

To validate the integration of the ocular model with the existing CVS model, we performed human experiments in the laboratory environment to measure cardiovascular (arterial pressure, heart rate, stroke volume, and cardiac output) and ocular (IOP) parameters during short-term (10 min) 6° HDT from 80° head-up tilt (HUT). Once validated, the multiscale model is used to investigate the acute cerebrovascular and ocular response to 6° HDT and to understand their interaction and potential effects on ocular changes. Specifically, we investigate the cerebrovascular dynamics elicited by posture change from 80° HUT to 6° HDT, observing the blood pressure and flow rate response at different levels of the brain (from proximal arterial to distal, capillary, and venous cerebral sites). Our analysis informs the mechanisms determining ICP and cephalic blood volume responses, thus contributing to the understanding of the relationship between cerebral hemodynamics and ocular responses (IOP, ocular globe volume) under altered-gravity. We present the model results concerning TLP and OPP during HDT and we discuss their role as potential risk factors for SANS. Our modeling framework is a promising tool to investigate cerebrovascular dynamics in altered-gravity, and results for our modeling efforts inform the pathophysiology of SANS and the development of future countermeasures.

## Results

In the following paragraphs, we first compare the model outcomes with our experimental ocular and cardiovascular measures. Then, we further explore detailed model predictions for the ocular-cerebrovascular response to acute 6° HDT.

### Model validation: experimental vs. modeled cardiovascular and ocular parameters

**Fig. 1:**
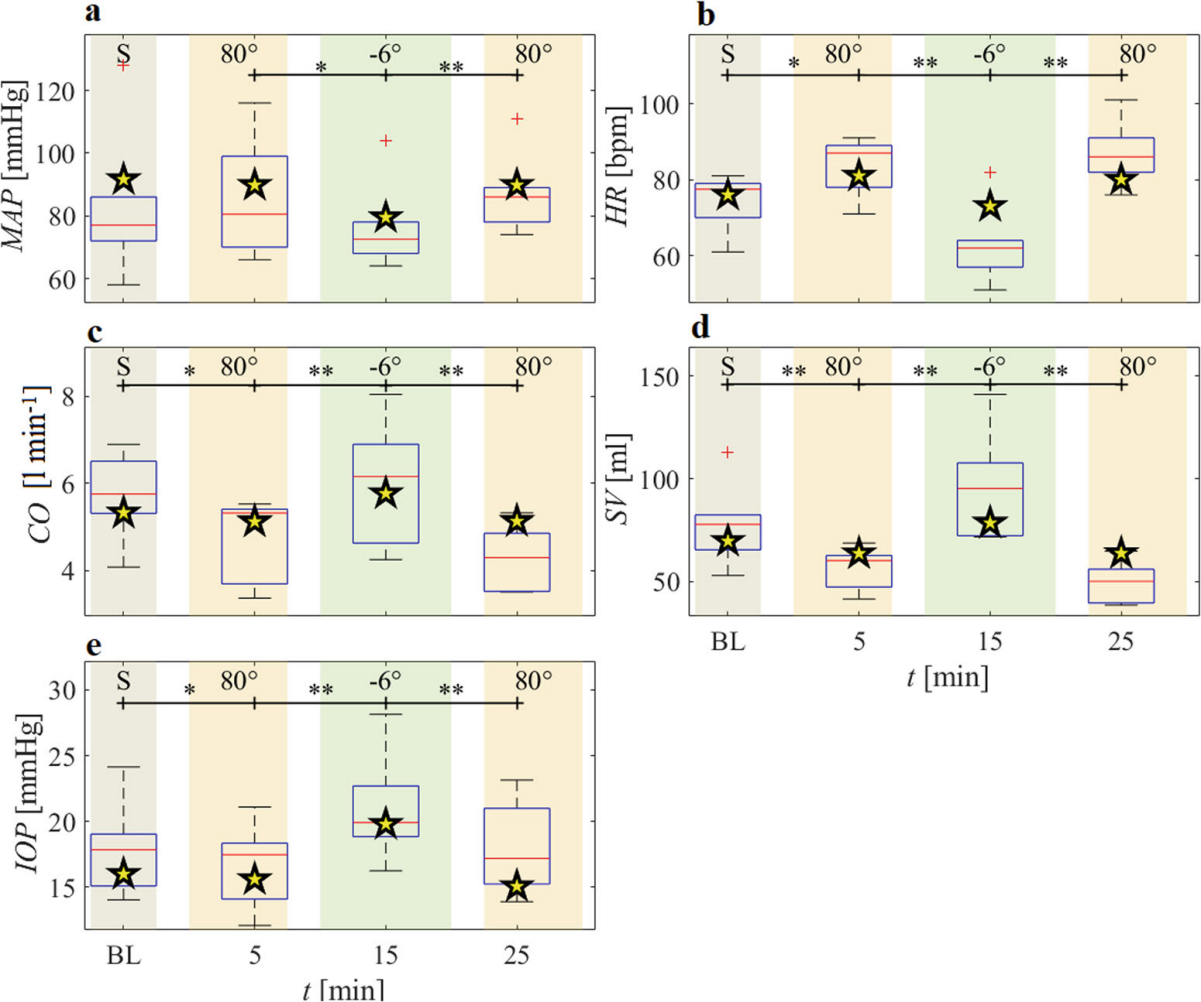
Experimental data and computational results. Cardiovascular and ocular experimental data (reported as boxplots, where the 2nd-3rd quartile range delimits the box, red lines mark distribution medians, and outliers are depicted as red crosses) compared to the corresponding model outcomes (yellow stars) at each phase of the HDT experiment. S denotes the baseline (BL) seated posture. MAP **a** indicates finger mean arterial pressure corrected at heart/brachial level, HR **b** indicates the heart rate, CO **c** indicates the cardiac output, SV **d** indicates the stroke volume, and IOP **e** indicates the intraocular pressure. Time *t* starts when subjects are first positioned upright (80° HUT) on the tilt table. Asterisks denote significant (*: *p* < 0.10, **: *p* < 0.05) parameter differences in the experimental data between tilt conditions.

Experimental data and corresponding model outcomes of the cardiovascular and ocular parameters obtained during the tilt maneuvers are displayed in Fig. 1. *p*-values (see Supplementary material) are computed via the Wilcoxon test for paired samples (*n* = 6 healthy, male subjects) on the experimental data between different postures. Subjects recruiting was conducted by selecting volunteers with anthropometric features as homogeneous and comparable as possible to those on which the model is calibrated (see Table 1).

**Table 1:** Subject characteristics

ID	age [years]	weight [kg]	height [cm]	BSA [m^2^]
M01	20	77	179	1.954
M02	21	82	177	1.990
M03	28	79	170	1.905
M04	26	78	179	1.967
M05	33	85	172	1.962
M06	31	65	180	1.828
Total	26.5 ± 5.2	77.6 ± 6.8	176.2 ± 4.2	1.934 ± 0.059
Model	25	75	175	1.900

Demographic and anthropometric characteristics of subjects enrolled in the experimental study (*n* = 6 males, mean ± SD) and the generic subject simulated by the CVS model. BSA is body surface area computed according to Du Bois method^84^.

The tilt maneuver from 80° HUT to 6° HDT causes an important headward fluid shift from the lower to the upper body^21,23^. The augmented cardiac filling leads to the rise in CO (Fig. 1c) and SV (panel d), accompanied by a drop in HR (panel b) and a slight decrease in MAP (panel a), modulated by the short-term regulation mechanisms. The model predictions for the post-tilt 80° HUT position are similar to the corresponding pre-tilt values for all parameters. IOP (panel e, both in vivo and modeled) follows the same trend as CO and SV, showing an acute rise of about +4.2 mmHg when tilting from 80° HUT to 6° HDT.

For all parameters, pre- and post-tilt 80° HUT positions are non-significantly different from each other (p > 0.05). Conversely, most parameters show statistically significant differences between pre- and post-tilt 80° HUT and 6° HDT positions (*p* < 0.05; with only MAP *p* < 0.10 between pre-tilt 80° HUT and 6° HDT). Exact *p*-values reported in Supplementary Table 8 in the Supplementary Material.

Results reported in Fig. 1 show that the model is able to accurately reproduce the in vivo ocular and cardiovascular response to the tilt maneuvers between 80° HUT and 6° HDT: (i) at 6° HDT the value predicted by the model falls inside the corresponding boxplot (CO, SV e IOP) or just outside it (MAP), with the only exception being HR, which experiences a reduced variation but in the same direction as experimentally observed; (ii) considering that pre- and post-tilt 80° HUT positions are non-significantly different for any of the parameters, also at 80° HUT the model prediction falls within or very close to the boxplot for all the analyzed CVS parameters. At fixed posture, relative errors of the value predicted by the model with respect to the mean value of the experimental data are within 15% for all the parameters (MAP: 80° HUT + 4.02%, 6° HDT + 3.92%; HR: 80° HUT –5.76%, 6° +15.87%; CO: 80° HUT + 13.04%, 6° HDT –4.31%; IOP: 80° HUT –11.66%, 6° HDT –5.56%), with the only exception of SV (80° HUT + 19.41%, 6° –19.20%).

Blomqvist et al.^22^ reported HR = 68 ± 2 bpm, CO = 7.4 ± 0.5 l min^−1^, SV = 108 ± 7 ml, systolic arterial pressure SAP = 104 ± 3 mmHg, and diastolic arterial pressure DAP = 59 ± 6 mmHg (diastolic) after a 30-min tilt to 5° HDT (n = 10 male individuals, baseline supine data: HR = 75 ± 4 bpm, CO = 7.7 ± 0.4 l min^−1^, SV = 102 ± 7 ml, SAP = 106 ± 3 mmHg, and DAP = 65 ± 2 mmHg). Shiraishi et al.^21^ performed a 6° HDT and compared the observed findings with baseline seated measures (*n* = 10 male individuals). They found that MAP decreased by 9 mmHg, HR decreased by 11 bpm, and CO increased by 0.7 l min^−1^. Linder et al.^59^ reported an IOP increase of approximately 3.5 mmHg on 10 individuals when tilting from the upright 90° position to 6° HDT. Laurie et al.^27^ reported a smaller IOP increase of 0.7 mmHg when 8 male subjects were tilted to 6° HDT, this time compared to a seated position.

### Ocular-cerebrovascular model results

In the following paragraphs, we first explore the blood pressure and flow rate model response to 6° HDT from 80° HUT throughout the cerebrovascular and ocular compartments. Then, we focus on and discuss the ocular-cephalic pressure and volume response during the tilt maneuver.

**Fig. 2:**
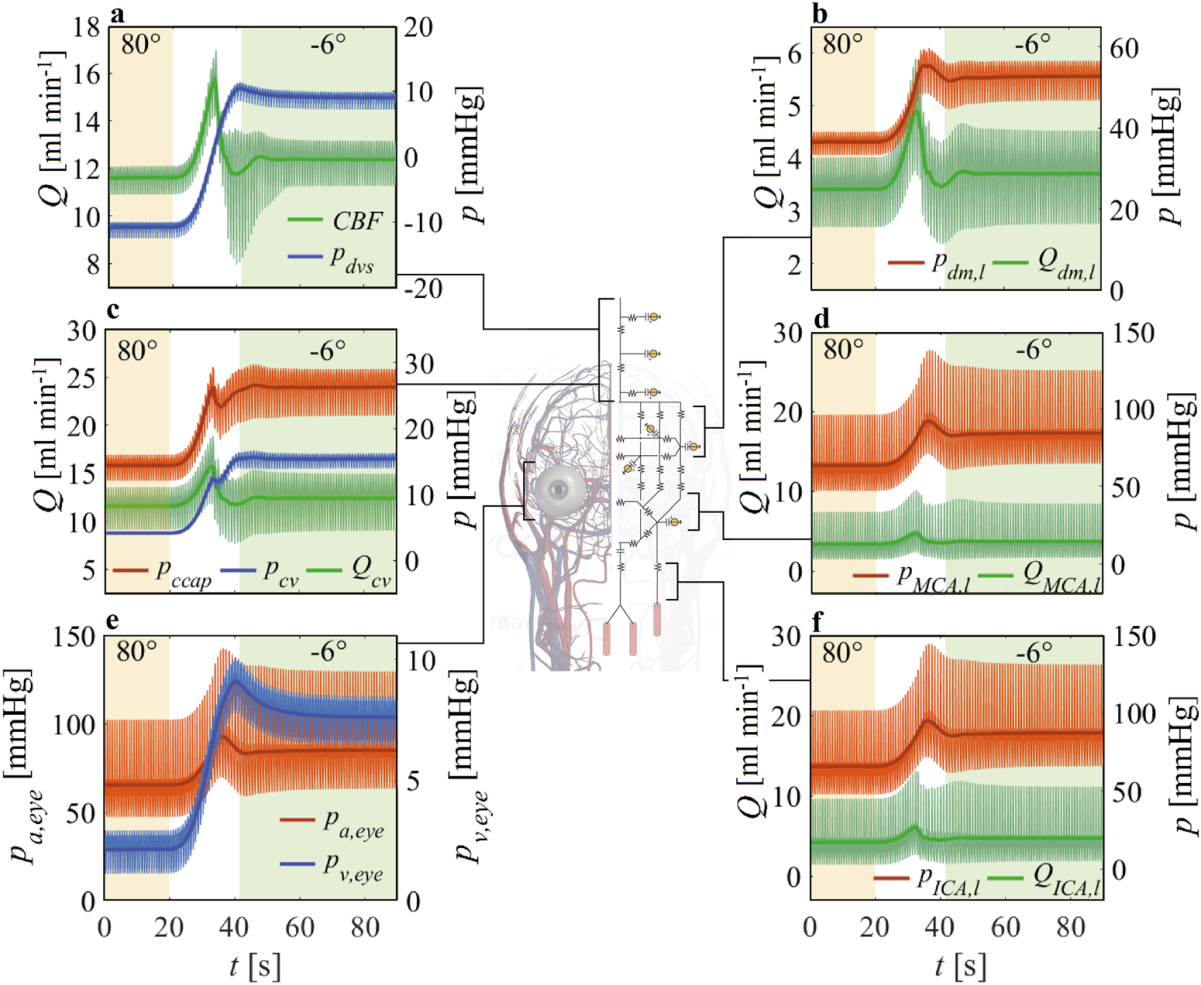
Ocular-cerebrovascular pressure and flow rate time responses to head-down tilt. Ocular and cerebrovascular time responses during a head-down tilt maneuver from 80° HUT to 6° HDT. Symbol p denotes pressure, Q is flow rate, and t is time. CBF and pdvs **a** indicate cerebral blood flow rate and dural venous sinus pressure, respectively. pdm,l and Qdm,l **b** indicate left distal middle cerebral artery blood pressure and flow rate. pccap, pcv, and Qcv **c** indicate cerebral capillary and venous pressures and cerebral venous flow rate, respectively. pMCA,l and QMCA,l **d** indicate left middle cerebral artery blood pressure and flow rate. pa,eye and pv,eye **e** indicate arterial and venous pressure at the level of the eye, while pICA,l and QICA,l **f** indicate left internal carotid artery blood pressure and flow rate, respectively. Thick lines represent the beat-averaged signals of the corresponding time series, which are depicted with thin lines.

Adopting a proximal-to-distal approach, Fig. 2 shows flow rate and pressure signals at the left (subscript l) internal carotid artery (ICA, panel f), the left middle cerebral artery (MCA, d), the left distal middle cerebral artery (dm, b), the cerebral capillary (ccap, c), cerebral venous (cv, c), and dural venous sinus (dvs, a) sites. In all instances, blood pressure rises at the beginning of the 6° HDT due to the change in the hydrostatic pressure.

The arterial and venous pressures at the level of the eye (Fig. 2e) show similar behavior to the cerebral arterial and distal-capillary-venous compartments in response to 6° HDT. The mean eye arterial pressure (pa,eye) rises by 19.6 mmHg (+30%) when tilting from 80° HUT to 6° HDT; that is less than the mean pressure increase detected at cerebral arterial level (+21.4 mmHg, +33%, at left ICA). This different change is due to the position of the eyes with respect to the body’s mid-coronal plane. As a result, we find a lower eye arterial pressure while the body is close to the horizontal position, given the more elevated position of the eye with respect to the rest of the cerebral circulation. Upon tilting from 80° HUT to 6° HDT, the mean eye venous pressure rises by about 5.5 mmHg (+260%).

Blood flow rates in all cerebrovascular compartments (Fig. 2f, d, b, and c, respectively) present a much more homogeneous response to the tilt maneuver from 80° HUT to 6° HDT compared to the pressure response. The first initial increase in flow rates detected at all levels at the beginning of the tilt maneuver—due to the sudden fluid shift from the lower to the upper body—is rapidly settled down to almost the same pre-tilt flow rate level shortly after approaching the 6° HDT position. This is due to the cerebral autoregulation acting at the distal level, rising cerebral distal resistances (+13%), and reducing the corresponding compliances (–17%) such that a nearly constant cerebral blood flow (CBF) is maintained^33^. Indeed, panel a of Fig. 2 displays CBF, which—after a wide positive overshoot during the HDT maneuver with a beat-averaged flow rate peak at 15.7 ml s − 1 ( + 35% compared to 80° HUT pre-tilt)—reaches again steady state at 12.4 ml −1 s (+6.6% with respect to 80° HUT pre-tilt) due to the cerebral autoregulation mechanism. Additional information on pressure and flow rate peak values are reported in Supplementary Table 9 in the Supplementary Material.

**Fig. 3:**
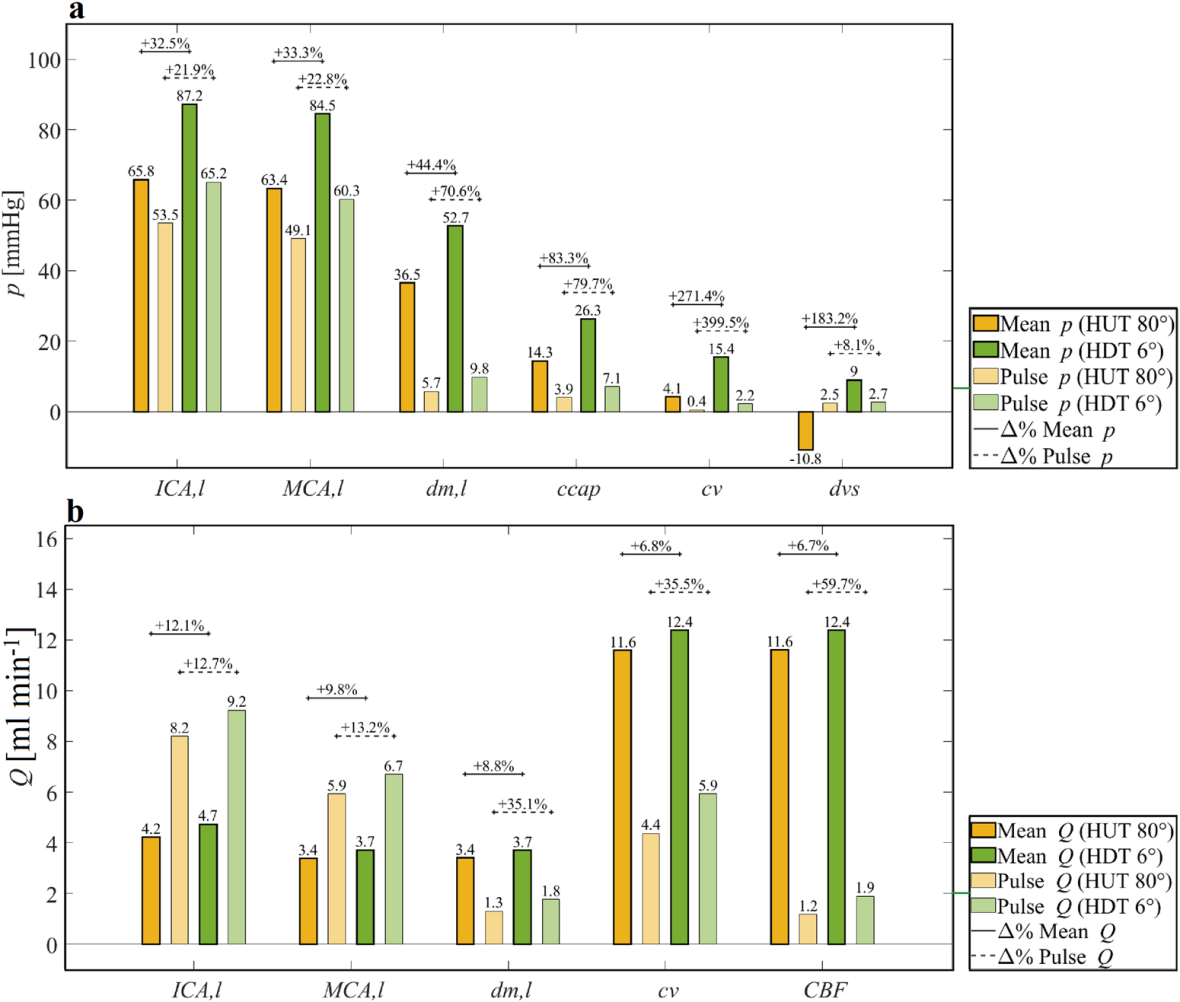
Cerebrovascular mean and pulse pressure and flow rate steady-state responses to head-down tilt. Pressure (**a**) and flow rate (**b**) mean and pulse responses throughout the cerebrovascular compartment during an HDT maneuver. For simplicity, only the left (l) side of the brain is included. ICA, MCA, dm, ccap, cv, and dvs indicate internal carotid artery, middle cerebral artery, distal middle artery, cerebral capillary, cerebral veins, and dural venous sinus compartments, respectively. CBF indicates cerebral blood flow rate. Mean (dark-colored bars) and pulse (max–min, light-colored bars) pressure and flow rate values are reported for all compartments during steady-state pre-tilt 80° HUT (80°HUT, yellow bars) and steady-state post-tilt 6° HDT (6° HDT, green bars). Percentage variation between pre- and post-tilt values is referred to as the 80° HUT values.

In Fig. 3 we summarize the steady-state mean and pulse (max–min) pressures and flow rates before and after the HDT maneuver at each cerebrovascular site (left ICA, MCA, dm, ccap, cv, and dvs), including the changes (in %) between 80° HUT and 6° HDT.

The head-down position causes blood pressure to increase at all levels of the cerebral vasculature^32^, reaching values comparable to the analogous vascular districts (e.g., arterial, capillary, venous) of the body in the same posture. For instance, mean pre-tilt (80° HUT) ICA,l pressure is about 65.8 mmHg (aortic MAP about 92.8 mmHg), whereas post-tilt (6° HDT) mean ICA,l pressure is about 87.2 mmHg, much closer to the post-tilt aortic MAP of about 86.1 mmHg.

Interestingly, the (mean) pressure variation following the HDT maneuver is not homogeneous throughout the observed cerebrovascular sites. Mean pressure increments after the HDT maneuver from 80° HUT to 6° HDT in the different cerebrovascular regions (from proximal to distal and capillary-venous regions) are as follows: +21.4 mmHg (+32.5%) at ICA,l, +21.1 mmHg (+33.3%) at MCA,l, +16.2 mmHg (+44.4%) at dm,l, +12 mmHg (+83.3%) at ccap), and +11.3 mmHg (+271.4%) at cv. Thus, going from proximal to distal/capillary-venous cerebral compartments, while the absolute pressure differences between 80° HUT and 6° HDT decrease, the corresponding relative (i.e., percentage) pressure differences increase. This could be explained by the reduction of the distal (pial) arteriolar compliances promoted by cerebral autoregulation to counteract the sudden CBF increase caused by head-down tilt, together with the non-linear behavior of the cerebral venous compliance (inversely related to the cerebral venous pressure).

The mean dvs pressure at the outflow of the cerebrovascular compartment changes from –10.8 mmHg at 80° HUT to 9 mmHg at 6° HDT ( + 19.8 mmHg, similarly to left ICA pressure), that is a 183.2% increase. This mean pressure increase differs from the previous trend of increase registered from ICA to cv compartments likely because of the influence of downstream superior vena cava (svc) pressure (mean values –0.5 mmHg at 80° HUT, 7.9 mmHg at 6° HDT) and central venous pressure CVP (2.1 mmHg at 80° HUT, 7.6 mmHg at 6° HDT). Indeed, it can be observed that the mean 80° HUT dvs pressure is given by subtracting the hydrostatic pressure related to the svc-head distance (about 10 mmHg at 80° HUT for a fluid column assumed to be 13.5 cm high) from the corresponding 80° HUT svc pressure.

Pulse pressure (Fig. 3a) tends to increase at all sites when tilting from 80°HUT to 6° HDT, reflecting the global pulse pressure increase registered at the central (aortic) level^47,60^. Furthermore, the change observed in pulse pressure is lower in relative (percentage) terms for the proximal arterial sites (ICA,l: +11.7 mmHg, +21.9%; MCA,l: +11.2 mmHg, +22.8%), whereas it is progressively higher for the downstream distal and capillary-venous sites (from +4.1 mmHg, +70.6% at dm,l to +1.8 mmHg, +399.5% at cv). The response of pulse pressure to HDT should be considered in light of the cerebral autoregulation contribution, which decreases the cerebral (arteriolar) blood compliance (together with the diminished cerebral venous compliance, inversely related to cv pressure), and thus further augments pulse pressure amplitude. Pulse pressure at dvs site is not very affected by head-down tilt (+0.2 mmHg, +8.1%), which is similar to the pulse pressure response of svc (+0.2 mmHg, +8.1%) and CVP ( + 0.2 mmHg, +12.5%).

Flow rate mean values (Fig. 3b) across the different cerebral compartments share similar responses to 6° HDT. Upon tilting, cerebral autoregulation acts such that the mean blood flow rate is maintained at nearly constant levels^32,33,61^. This especially happens at distal sites (dm,l)—directly controlled by autoregulation—showing very limited (+0.3 ml, −1 + 8.8%) mean flow rate variations following 6° HDT. The adjacent sites show a similar response, limiting the amount of mean flow rate variation at 6° HDT (mean flow rate at ICA,l: +0.5 ml s − 1, +12.1%; MCA,l: +0.3 ml s − 1, +9.8%; cv: +0.8 ml s − 1, +6.8%). As a result, CBF is well maintained during posture change from 80° HUT to 6° HDT^33^, with +0.8 ml s − 1 ( + 6.7%) mean flow rate variation. On the other hand, flow rate pulse values (Fig. 3b) increase along the proximal to distal/capillary-venous direction (ICA,l: +12.7%, MCA,l: +13.2%, dm,l: +35.5% and CBF: +59.7%), similarly to corresponding pulse pressures.

**Fig. 4:**
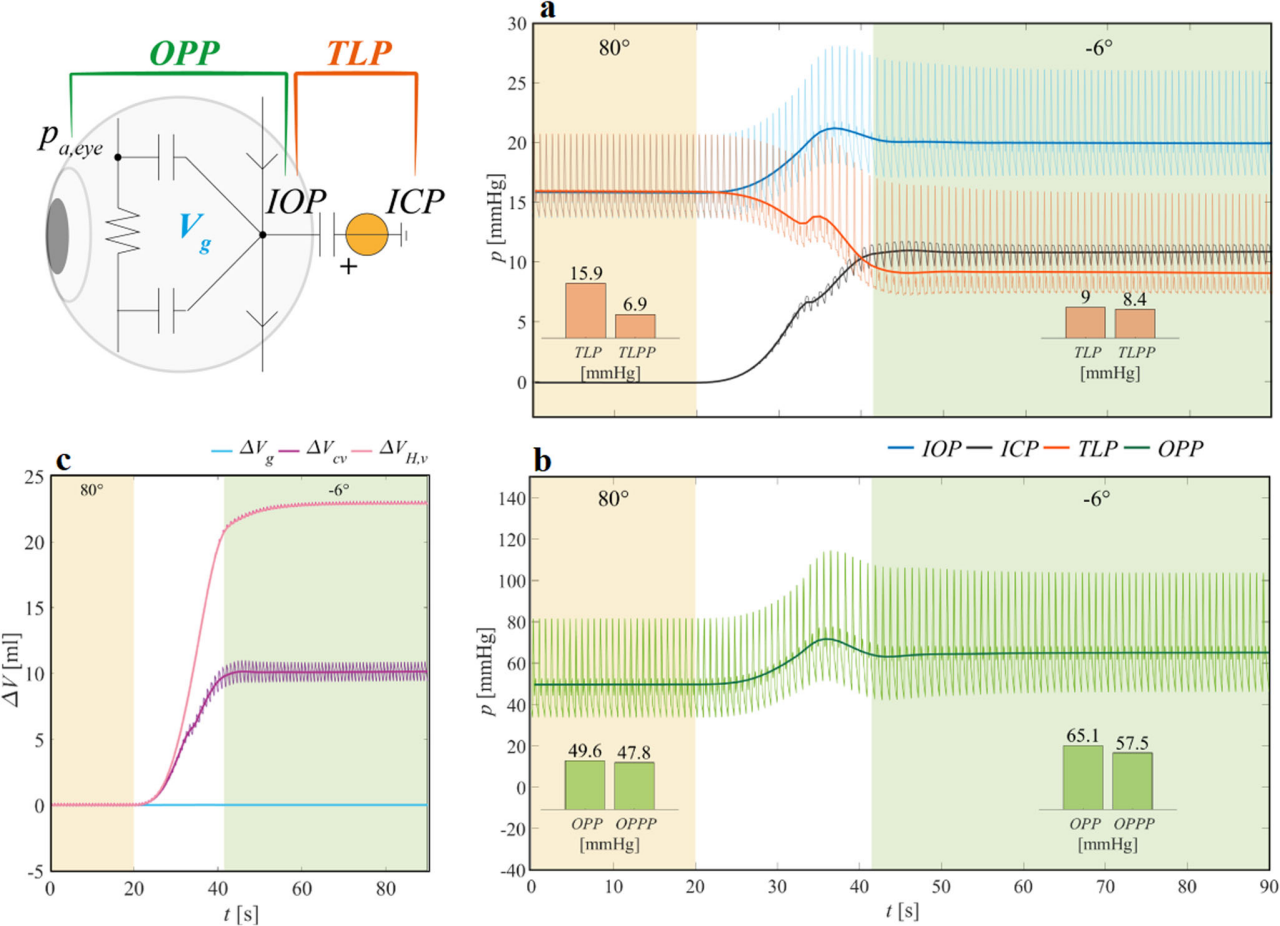
The ocular-cephalic compartment time responses during a head-down tilt maneuver from 80° HUT to 6° HDT. Panel **a**: intraocular (IOP), intracranial (ICP), and translaminar (TLP) pressures. Panel **b**: ocular perfusion pressure (OPP). Panel **c**: ocular globe (∆Vg), cerebral veins (∆Vcv), and extra-cerebral head veins (∆VH,v) changes in blood volume. In the insets of panels **a**, **b**, TLP and OPP mean and pulse (TLPP, OPPP) pressure values before and after the tilt maneuver are also reported. pa,eye indicates eye arterial pressure, t denotes time, and ∆*V* indicates the absolute volume variation with respect to the initial 80° HUT value.

To elucidate the role of posture changes on the ocular apparatus, in Fig. 4 we focus on the corresponding ocular model compartment, highlighting its interplay with the cerebrovascular circulation. The relationship between IOP and ICP (i.e., the translaminar pressure TLP = IOP − ICP), as well as between IOP and the arterial pressure at eye level (ocular perfusion pressure OPP = pa,eye − IOP) might play a crucial role in the development of SANS. Thus, here we investigate their responses during the acute phase of HDT^13–15,25,27^.

Figure 4a shows IOP and ICP response to 6° HDT. When tilting from 80° HUT to 6° HDT, ICP increases from a few negative (–0.1) mmHg to about 10.9 mmHg, whereas IOP changes from 15.8 mmHg to 19.9 mmHg (in line also with most IOP measurements in actual microgravity^36,37,39,40^). Lawley et al.^13^ reported ICP to increase from 4 ± 1 mmHg to 14 ± 2 mmHg when tilting subjects from 90° HUT (seated) to 0° supine. ICP increased even further (an additional +1.8 ± 0.5 mmHg) when tilting from 0° supine to 6° HDT. Holmlund et al.^62^ found ICP to increase from –0.9 ± 3.5 mmHg at 69° HUT to 10.4 ± 1.5 mmHg at 0° supine. In all cases, ICP seems to increase by approximately 10/12 mmHg when changing posture from upright to supine/head-down. In addition, we find that pulse ICP rises from 0.01 mmHg (80° HUT) to 1.7 mmHg (6° HDT), while pulse IOP increases from 6.9 mmHg (80° HUT) to 8.7 mmHg (6° HDT).

Thus, both mean IOP and ICP increase after the head-down tilt maneuver, as a consequence of the fluid shift to the upward-cephalic compartments together with the increase in blood pressure at the eye and cerebral level. Our modeling efforts show that ICP increases more than IOP following HDT, causing mean TLP to decrease from the pre-tilt value of 15.9 mmHg to the post-tilt value of 9 mmHg. This decrease of about –43% in TLP—albeit our analysis only takes into account the acute response to HDT—is deemed to represent one potential contributor to SANS development in astronauts^13,14^. Despite the observed beat-averaged decrease in TLP following 6° HDT, pulse (max–min) TLP (TLPP) widens by +1.5 mmHg (from 6.9 mmHg to 8.4 mmHg, +22%) after 6° HDT as a result of the augmented pulse IOP and ICP.

Figure 4b reports the OPP response during a head-down tilt maneuver from 80° HUT to 6° HDT. Differently from TLP, beat-averaged OPP goes up from 49.6 mmHg to 65.1 mmHg (+31%) as a consequence of the augmented arterial pressure at eye level (+19.6 mmHg, Fig. 2e) overcoming the more limited increase of IOP ( + 4.1 mmHg, Fig. 4a). The higher OPP found at the head-down position reveals an increasing perfusion pressure along the outside-inside globe direction^17^, potentially leading to optic disk edema^31^. As already underlined for TLPP, also pulse (max–min) OPP (OPPP) increases upon reaching 6° HDT by +9.7 mmHg (from 47.8 mmHg at 80° HUT to 57.5 mmHg at 6° HDT, +20%).

Due to SANS association with cephalic volume congestion deriving from headward fluid shift^9–13,15^, we used the model to predict the ocular-cerebrovascular volume variations in response to 6° HDT. In Fig. 4c we report the absolute volume differences with respect to the pre-tilt 80° HUT state for the eye globe (Vg), the cerebral veins (Vcv), and the extra-cerebral head veins (VH,v) during HDT. Figure 4c shows that, despite almost no volume change occurring to the ocular globe, both cerebral and extra-cerebral venous volumes are highly affected by the headward fluid shift, increasing from the upright values due to the HDT. Thus, although IOP increases due to HDT, the volume of the eye globe stays nearly unchanged, appearing to be insensible to posture variations (∆Vg ≃ +0.005 ml after HDT). Conversely, the marked cephalic congestion highlighted by the cerebral and extra-cerebral blood volume increases underlines how the retrobulbar space can exert higher compression forces on the ocular globe and, over prolonged time, may induce structural remodeling^7^.

## Discussion

Given the importance of understanding the mechanisms leading to SANS development in astronauts during long-term space missions^5^, in the present work, we investigate the ocular-cerebrovascular interaction elicited by acute 6° HDT as an analog of microgravity. To this end, we used our in silico-in vivo validated framework described in this study. The understanding of the posture-induced systemic, cerebral, and ocular responses to 6° HDT contributes to identifying the main potential risk factors leading to visual disorders observed in the literature^7^.

As the body is tilted head-down, the headward fluid shift from the lower extremities causes an increasing accumulation of blood volume in the cephalic compartments (Fig. 4c). Upon reaching 6° HDT, blood pressure in the cerebral (and extra-cerebral) compartments increases, with mean pressure at left ICA increasing by +32.5%, at left distal level by +44.4%, and at cerebral veins by +271.4%, with respect to pre-tilt 80° HUT values (Figs. 2b–d, f and 3a). The mean pressure increase following tilt down to 6° HDT is related to the hydrostatic pressure of the blood column extending from the head to the central level, which is about 27 mmHg at the right and left ICA level, at 80° HUT (please, refer to 1D arterial geometry data reported in Supplementary Table 1 in the Supplementary Material). The proximal-to-distal/capillary-venous trend of increase shown for the mean pressure relative difference between pre- and post-tilt to 6° HDT can be explained by the decreased cerebral arteriolar compliance dictated by cerebral autoregulation and by the decreased cerebral venous compliance (inversely related to cerebral venous pressure). Therefore, distal and capillary/venous cerebral vessels become stiffer and less compliant when approaching the head-down position. Pulse pressure in the cerebral circulation shows a similar behavior (Figs. 2b–d, f and 3a), with an overall increase reflecting the global augmented pulse pressure found at the central (aortic) level, although to a larger extent (aortic pulse pressure rises by +17% going from 80° HUT to 6° HDT), combined with a trend towards increase from proximal to distal/capillary-venous levels elicited by the reduced distal and venous cerebral compliance.

Blood flow rates are affected only to a limited extent by 6° HDT (Figs. 2a-d, f and 3b), with almost unchanged mean flow rate levels at all cerebral compartments, due to the presence of cerebral autoregulation acting to preserve adequate cerebral perfusion. At the same time, pulse flow rate is overall enlarged, and shows a trend to increase along the proximal-to-distal/capillary-venous direction. This outcome results as a magnification of the increased flow rate pulsatility detected at the aortic level (+8% from 80° HUT to 6° HDT) and the reduced distal/venous cerebral compliance.

With our modeling approach, we provide insights into the pulsatility of pressure and flow rate signals, which are difficult to measure in vivo and thus generally unexplored in the literature, especially concerning cerebral circulation. Yet, the higher pressure and flow rate signals pulsatility detected at 6° HDT implies that short-term HDT causes the cerebral circulation (particularly in the distal districts) to be subject to higher and lower pressure/flow rate values compared to the beat-averaged levels, leading to an overall increased risk of mechanical stress and cerebrovascular damage^63,64^.

The computational model is also useful to explore the role of different hemodynamic terms shaping ICP and IOP response to 6° HDT. The ICP behavior during 6° HDT (Fig. 4a) results from the combined effect of all pressure changes detected within the cerebrovascular compartment (Fig. 3a), albeit the major contributor to this change comes from the hydrostatic pressure of the cerebrospinal fluid. Considering the +11 mmHg change in ICP when tilting from 80° HUT to 6° HDT, the majority of this change (90%, about 10 mmHg) comes from the hydrostatic column of cerebrospinal fluid. Thus, the hydrostatic pressure linked to fluid weight and body posture is the main contributor to determining ICP response to HDT. In addition, pulse ICP shows a modest absolute variation (+1.7 mmHg) approaching 6° HDT as a consequence of the overall increased pulse pressure of the communicating cerebral compartments and due to the nature of the intracranial compliance (inversely related to ICP). IOP depends instead on the arterial and venous blood pressure at the level of the eye, as well as on ICP, aqueous humor dynamics, globe volume, and ocular compliances. Nevertheless, our model confirms that, among all contributions, arterial and venous pressure at eye level (Fig. 2e) are those exerting the highest influence on IOP through posture-induced hydrostatic pressure variation^15,65^, at least in the acute HDT phase. Also, pulse IOP reflects the pulse pressure increase of its determining factors, that is blood pressure at eye level in particular.

TLP decreases as a consequence of the posture-driven increases in mean IOP and ICP revealed at 6° HDT. However, the limited increase in IOP compared to the much larger increase in ICP leads to an evident alteration (i.e., decrease) of the differential pressure between the eye and the retrobulbar space (TLP)^25,27^. In addition, the difference between the arterial pressure at eye level and IOP (OPP) is also altered (i.e., increased)^31^. The model predicts a –43% decrease for TLP and a + 31% increase for OPP following tilt down to 6° HDT (Fig. 4). Therefore, as hypothesized by several authors^13,14,25,28,66^, we show that the normal equilibrium between the eye globe and the retrobulbar space (TLP), as well as the perfusion state (OPP) of the eye may be transiently impaired when adopting a strict 6° HDT position^13,31^. Moreover, depending on the pre-tilt subjects’ ICP (which can vary between different individuals), TLP may even become negative^14^, with a pressure outside the eye globe higher than inside the globe. These conditions could set the base for ocular anatomical remodeling, structural changes, edema formation, and other visual disorders, especially in the case of chronic exposure^13^ and over longer time scales^14^.

IOP and ICP have also been shown to be susceptible to other factors such as ambient CO2 levels^9,13,15,27^ or body and tissue weight^67,68^, which are not simulated by our model. Given the role of CO2 as a cerebrovascular vasodilator promoting ICP and IOP increase^9,13,15,27^, this parameter should be accounted for in future HDT numerical investigations and represents thus a limitation of our current model. Additionally, body and tissue weight—completely removed in actual 0 g conditions^67,68^ though not accounted for in our modeling layout—should be included in the modeling framework as an additional posture-dependent parameter influencing vascular and ocular variables during HDT^36,69,70^. Moreover, while HDT is a reasonable microgravity model for many applications, it is important to acknowledge the physiological differences between HDT and true microgravity. One key difference is related to CVP, which is found to increase in HDT, as shown in our simulations, although it decreases in true microgravity^71^. However, the main limitation of our model at present relates to its applicability to acute 6° HDT exposure. In particular, long-term regulation mechanisms for renal and hormonal activity, autoregulation of blood and aqueous humor dynamics, and loss of circulating blood volume enhanced by capillary filtration (from intravascular volume to interstitial space) are not included in our computational framework. Therefore, the implementation of such long-term mechanisms represents a future improvement of our modeling framework to investigate long-duration microgravity-driven effects on IOP and ICP^15–17,72^. Last, the reduced sample size (*n* = 6 males) is a limiting aspect of the present work, and including more subjects (also females) would provide a more accurate CVS modeling validation in different postures.

In conclusion, our numerical framework can accurately mimic the ocular and cardiovascular response to acute 6° HDT. Despite the limitations discussed above, our model is a promising tool to investigate the complex physiological mechanisms contributing to the development of SANS, also by accounting for patient- and gender-specific differences in human spaceflight crews. Modeling approaches can support the identification of key mechanisms leading to the development of SANS, informing the development of new countermeasures. These include, but are not limited to lower-body negative pressure^73,74^, thigh cuffs to reduce cephalic fluid shift^16,75,76^, and mechanically induced increased IOP (e.g., through swimming goggles^16,77^), to mitigate cerebrovascular and ocular alterations related to long-term space missions.

## Methods

### Mathematical model

**Fig. 5:**
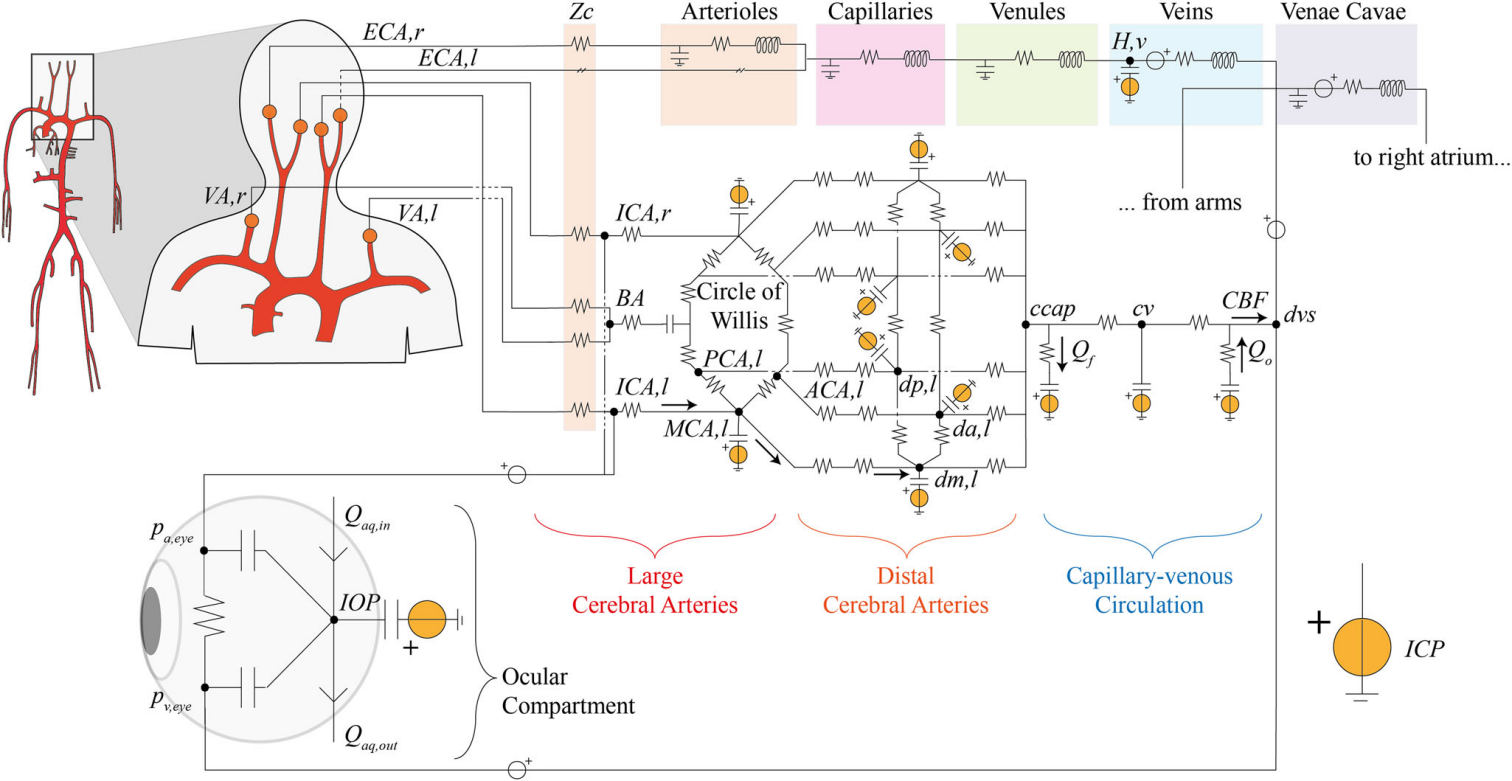
CVS model architecture: focus on the cerebrovascular and ocular compartments. The 1D arterial network (red vessels, the upper aortic-vertebral, and carotid branches are reported here as a magnification of the entire arterial network, on the left) is connected to the 0D lumped-parameter head, cerebral, and ocular compartments (Zc denotes lumped characteristic impedances). ECA,l, and ECA,r represent the left and right external carotid arteries, connected to the extra-cerebral head circulation, where H,v denotes (extra-cerebral) head veins. Left and right internal carotid arteries are denoted as ICA,l and ICA,r while VA,l and VA,r represent the left and right vertebral arteries, respectively (BA indicates the basal artery). For the left (l) cerebrovascular circulation, ACA, MCA, and PCA indicate the anterior, middle, and posterior large (proximal) cerebral arteries, while da, dm, and dp denote distal anterior, middle, and posterior arteries, respectively. The right (r) cerebral circulation is symmetric to the left side and not represented here for simplicity. Compartments ccap, cv, and dvs refer to the cerebral capillaries, cerebral veins, and dural venous sinus, respectively. Qf and Qo are the cerebrospinal fluid formation and outflow rates, respectively, while CBF represents the cerebral blood flow rate and ICP denotes the intracranial pressure. In the ocular compartment, IOP denotes the intraocular pressure, whereas pa,eye, pv,eye, Qaq,in, and Qaq,out indicate the arterial and venous pressures at the level of the eye and the aqueous humor inflow and outflow rates, respectively.

The adopted numerical framework is a closed-loop, multiscale model of the human cardiovascular system (CVS), combining a 1D representation of the arterial tree with multiple 0D descriptions of the systemic peripheral, venous, cardio-pulmonary, coronary, and cerebrovascular circulations. The CVS model integrates our previously validated and published architectures^47,52,78^, and is calibrated on a healthy male individual with age 25 yo, weight 75 kg, and height 175 cm. Gravity is introduced in the CVS model equations to account for posture-induced effects, by considering the angle between the longitudinal body axis and the horizontal reference in a simulated tilt-table framework^47^. The model architecture is shown in Fig. 5. In the following paragraphs we provide a brief overview highlighting the most important functional aspects of the model. Additional model details, including the model equations, the parameter settings, and the adopted numerical method, are included in the Supplementary Material.

The 1D representation of the arterial tree includes 63 main large arteries schematized as a network of tapered vessels and bifurcations (top-left side of Fig. 5). Blood motion throughout 1D vessels is described by the axisymmetric form of Navier-Stokes equations for mass and momentum balance. The non-linear constitutive law adopted to mimic the mechanical behavior of the arterial wall relates blood pressure and the local cross-section area accounting for the visco-elastic properties of the vessel^79^.

Each 1D terminal branch (orange circles in Fig. 5) is connected to 0D electric analogs of the downstream circulation via a set of lumped characteristic impedances, Zc. Figure 5 illustrates in detail only the upper portion of the body (lower body not included for brevity), where the external carotid arteries blood flow goes into the extra-cerebral head circulation, further subdivided into an arterial, capillary, venular, and venous compartment. The blood flow coming from the internal carotids and vertebral arteries is now connected to the ocular-cerebrovascular circulation (this is the main modification with respect to the previous version of the model^78^). The cerebrovascular circulation combines the lumped-parameter model of the human eye proposed by Nelson et al.^51^ and Petersen et al.^31^ with the lumped-parameter model of the cerebral circulation developed by Ursino and Giannessi 2010^53^.

The lumped-parameter model of the eye is composed of six compartments governing the intraocular pressure (IOP) and the ocular globe volume (Vg), using the arterial and venous pressure at the level of the eye (pa,eye and pv,eye, respectively) as well as the intracranial pressure (ICP) coming from the cerebrovascular model, as inputs. The ocular arterial, venous, and globe compliances employed in the ocular model are non-linear and they are dependent on both IOP and Vg (the model is calibrated according to physiological values reported in refs. 80,81). The variables pa,eye and pv,eye, which are taken at the internal carotid and dural venous sinus level, respectively, include the effects of gravity changes during tilt by considering the vertical hydrostatic column between the globe and the mid-coronal plane (details in ref. 31).

The model of the cerebrovascular circulation is a lumped representation of the main large cerebral arteries of the circle of Willis, branching into the right and left pial circulation and intracerebral arterioles (subdivided into anterior, middle, and posterior distal compartments, communicating one another through cortical collateral vessels). The vessel compliances and hydraulic resistances of the pial circulation are controlled by the action of cerebral autoregulation (aimed at maintaining a nearly constant level of cerebral blood flow) and CO2 reactivity. A unique capillary-venous branch closes the cerebrovascular network. The formation and outflow dynamics of the cerebrospinal fluid and the hydrostatic contribution due to gravity regulate ICP via non-linear intracranial compliance (details in ref. 53 and in the Supplementary Material).

The outflow of the cerebrovascular model connects directly to the superior vena cava compartments, jointly with the arms region blood flow, the extra-cerebral head circulation, and the ocular model outflow (Fig. 5).

The remaining CVS model is organized by grouping the 1D terminal branches into four distinct body regions (head, arms, upper and lower abdomen, and legs), adopting the same lumped structure as for the 0D head extra-cerebral circulation (Supplementary Fig. 1 in the Supplementary Material), that is subdividing into arterioles, capillaries, venules, veins, and venae cavae). Venous valves are enclosed within arm and leg veins to prevent reverse blood flow. A specific representation of the coronary microvascular bed is attached to the 1D large coronary arteries^82^, whereas additional lumped parameterizations are adopted to model the four cardiac chambers, cardiac valves, and pulmonary circulation. The cardio-pulmonary compartments are subject to the action of the extra-vascular and extra-chamber intrathoracic pressure (ITP). Changes in the relative orientation between the body and the gravity vector (as during tilt) cause changes in ITP—simulating the motion of the diaphragm—contributing to the overall response to acute posture variations^78,83^.

The autonomic control systems include the baroreflex and cardio-pulmonary reflex mechanisms to regulate short-term pressure variations elicited by acute posture changes. They are modeled as detailed in refs. 49 and 47.

### Human experiments

Six healthy male volunteers were recruited to participate in the experiment. Subjects’ demographic and anthropometric characteristics are reported in Table 1, which also includes the CVS model characteristics used to calibrate the numerical framework. None of the subjects enrolled in this study had a history of previous, recent, or ongoing use of medications at the time of the study. All subjects were instructed to avoid intense exercise and caffeine within 12 h prior to the study. Subjects were appositely screened by completing a questionnaire in order to report a history of previous or ongoing cardiovascular or ocular conditions or any other exclusion criteria. All performed procedures involving human participants were approved by the Human Research Protection Program of Texas A&M University (Institutional Review Board number IRB2022-1070), and in accordance with the Declaration of Helsinki (1964) and its later amendments. Written informed consent was obtained from all participants included in the study.

The experiment consisted of one baseline session and one experimental session. During the baseline session, the subjects were in a seated position, and after 5 min of rest, a first set of cardiovascular measures (described below) was collected. During the experimental session, the subjects were positioned in a tilt table at 80° HUT. After 5 min of rest, another set of cardiovascular measures was collected. the subjects were then tilted to 6° HDT, and after 5 min of rest, a third set of cardiovascular measures was collected. The subjects spent a total of 10 min in a head-down position. They were then tilted back to 80° HUT, and one last set of cardiovascular measures was collected after 5 min. For safety reasons, the subjects were strapped to the tilt table for the entire duration of the session.

The cardiovascular measures included the following: (i) brachial arterial pressure (sphygmomanometer, Omron cuff) used for calibration, (ii) heart rate, cardiac output, and stroke volume (Innocor inert gas rebreathing integrated with a finger pulse oximeter, Cosmed), and (iii) intraocular pressure (eye tonometer, iCare). In addition, during both experimental sessions, subjects’ finger arterial pressure was recorded continuously using a Finometer NOVA (plethysmography, Finapres Medical Systems), corrected at brachial-heart level with the height correction unit, and periodically calibrated with the brachial pressure acquired through the sphygmomanometer (i). For each set of measures, the corresponding MAP value is computed by averaging the continuous pressure signal acquired by the Finometer NOVA over 1 min of recording, starting from immediately after the Innocor gas rebreathing (ii). Other measures—such as oxygen consumption and autonomic indices, blood flow velocity, and cross-section areas in the common carotid artery and internal jugular vein—were monitored and collected. Although not directly exploitable for modeling validation and therefore not shown here, all the performed recordings make each measurement session quite complex and rich. Additional information regarding the employed instrumentation, usage of devices, and environmental calibration procedure can be found in refs. 12,30.

Boxplots of all variables at all measurement time points were calculated. Data were non-normally distributed (*p* ∼ 1e-15, two-tailed Kolmogorov–Smirnov test for normality, *n* = 6). Statistical significance of parameter differences between time points has therefore been assessed via a non-parametric Wilcoxon test for paired samples (two-tailed, *n* = 6).

### Supplementary information

Manuscrpt Suplementay Material

## Data Availability

The datasets corresponding to the experimental data are available here: https://github.com/BHP-Lab/acute-midterm-hdbr.

## Code availability

The underlying code for the mathematical model will be made available by the corresponding author on reasonable request.

## References

[1] Gunga, H.-C. in *Human physiology in extreme environments*, chap. 7, 2nd edn. 273–311 (Academic Press, 2020).

[2] Young, L. R. & Sutton, J. P. *Handbook of Bioastronautics* (Springer, 2021).

[3] Blaber E, Marçal H, Burns BP (2010). Bioastronautics: the influence of microgravity on astronaut health. Astrobiology.

[4] Clément, G. *Fundamentals of Space Medicine*, Vol. 23 (Springer Science & Business Media, 2011).

[5] Laurie, S. S. et al. *Risk of Spaceflight Associated Neuro-ocular Syndrome (SANS)*. In: NASA Human Research Program—Human Health Countermeasures Element (National Aeronautics and Space Administration, Lyndon B. Johnson Space Center, Houston, Texas, 2022). URL https://humanresearchroadmap.nasa.gov/risks/risk.aspx?i=105.

[6] Patel ZS (2020). Red risks for a journey to the red planet:the highest priority human health risks for a mission to mars. npj Microgravity.

[7] Mader TH (2011). Optic disc edema, globe flattening, choroidal folds, and hyperopic shifts observed in astronauts after long-duration space flight. Ophthalmology.

[8] Marshall-Bowman K, Barratt MR, Gibson CR (2013). Ophthalmic changes and increased intracranial pressure associated with long duration spaceflight: an emerging understanding. Acta Astronaut..

[9] Bateman GA, Bateman AR (2022). A perspective on spaceflight associated neuro-ocular syndrome causation secondary to elevated venous sinus pressure. npj Microgravity.

[10] Lee AG (2020). Spaceflight associated neuro-ocular syndrome (sans) and the neuro-ophthalmologic effects of microgravity: a review and an update. npj Microgravity.

[11] Norsk P (2020). Adaptation of the cardiovascular system to weightlessness: surprises, paradoxes and implications for deep space missions. Acta Physiol..

[12] Whittle RS, Diaz-Artiles A (2023). Gravitational effects on carotid and jugular characteristics in graded head-up and head-down tilt. J. Appl. Physiol..

[13] Lawley JS (2017). Effect of gravity and microgravity on intracranial pressure. Physiol. J..

[14] Zhang L-F, Hargens AR (2018). Spaceflight-induced intracranial hypertension and visual impairment: pathophysiology and countermeasures. Physiol. Rev..

[15] Huang AS, Stenger MB, Macias BR (2019). Gravitational influence on intraocular pressure: implications for spaceflight and disease. J. Glaucoma.

[16] Otto, C. in *Handbook of Bioastronautics*, chap. 51, 641–673 (Springer, 2021).

[17] Taibbi G, Cromwell RL, Kapoor KG, Godley BF, Vizzeri G (2013). The effect of microgravity on ocular structures and visual function: a review. Surv. Ophthalmol..

[18] Gunga, H.-C., von Ahlefeld, V. W., Coriolano, H.-J. A., Werner, A. & Hoffmann, U. in *Cardiovascular System, Red Blood Cells, and Oxygen Transport in Microgravity*, chap. 2, 11–34 (Springer, 2016).

[19] Grigoriev A, Kotovskaya A, Fomina G (2011). The human cardiovascular system during space flight. Acta Astronaut..

[20] Watenpaugh DE (2016). Analogs of microgravity: head-down tilt and water immersion. J. Appl. Physiol..

[21] Shiraishi M, Schou M, Gybel M, Christensen NJ, Norsk P (2002). Comparison of acute cardiovascular responses to water immersion and head-down tilt in humans. J. Appl. Physiol..

[22] Blomqvist CG, Nixon J, Johnson RL, Mitchell JH (1980). Early cardiovascular adaptation to zero gravity simulated by head-down tilt. Acta Astronaut..

[23] Pavy-Le Traon A, Heer M, Narici MV, Rittweger J, Vernikos J (2007). From space to earth: advances in human physiology from 20 years of bed rest studies (1986–2006). Eur. J. Appl. Physiol..

[24] Ong J, Lee AG, Moss HE (2021). Head-down tilt bed rest studies as a terrestrial analog for spaceflight associated neuro-ocular syndrome. Front. Neurol..

[25] Marshall-Goebel K (2017). Intracranial and intraocular pressure during various degrees of head-down tilt. Aerospace Med. Hum. Perform..

[26] Prata TS, De Moraes CG, Kanadani FN, Ritch R, Paranhos A (2010). Posture-induced intraocular pressure changes: considerations regarding body position in glaucoma patients. Surv. Ophthalmol..

[27] Laurie SS (2017). Effects of short-term mild hypercapnia during head-down tilt on intracranial pressure and ocular structures in healthy human subjects. Physiol. Rep..

[28] Laurie SS (2019). Optic disc edema after 30 days of strict head-down tilt bed rest. Ophthalmology.

[29] Roberts D (2015). Structural brain changes following long-term 6 head-down tilt bed rest as an analog for spaceflight. Am. J. Neuroradiol..

[30] Whittle RS (2022). Gravitational dose-response curves for acute cardiovascular hemodynamics and autonomic responses in a tilt paradigm. J. Am. Heart Assoc..

[31] Petersen LG (2022). Gravitational effects on intraocular pressure and ocular perfusion pressure. J. Appl. Physiol..

[32] Du J (2021). Alterations in cerebral hemodynamics during microgravity: a literature review. Med. Sci. Monit. Int. Med. J. Exp. Clin. Res..

[33] Cooke WH, Pellegrini GL, Kovalenko OA (2003). Dynamic cerebral autoregulation is preserved during acute head-down tilt. J. Appl. Physiol..

[34] Marshall-Goebel K (2021). Mechanical countermeasures to headward fluid shifts. J. Appl. Physiol..

[35] Klein T (2020). Transient cerebral blood flow responses during microgravity. Life Sci. Space Res..

[36] Anderson AP (2016). Acute effects of changes to the gravitational vector on the eye. J. Appl. Physiol..

[37] Chung K-Y (2011). Diurnal pattern of intraocular pressure is affected by microgravity when measured in space with the pressure phosphene tonometer (ppt). J. Glaucoma.

[38] Draeger J, Schwartz R, Groenhoff S, Stern C (1993). Self-tonometry under microgravity conditions. Clinical Investig..

[39] Mader TH (1991). Intraocular pressure in microgravity. J. Clin. Pharmacol..

[40] Mader CTH (1993). Intraocular pressure and retinal vascular changes during transient exposure to microgravity. Am. J. Ophthalmol..

[41] Evensen KB, Eide PK (2020). Measuring intracranial pressure by invasive, less invasive or non-invasive means: limitations and avenues for improvement. Fluids Barriers CNS.

[42] Zhang X (2017). Invasive and noninvasive means of measuring intracranial pressure: a review. Physiol. Meas..

[43] Petersen LG, Petersen JCG, Andresen M, Secher NH, Juhler M (2016). Postural influence on intracranial and cerebral perfusion pressure in ambulatory neurosurgical patients. Am. J. Physiol. Regul. Integr. Comp. Physiol..

[44] Andresen M, Hadi A, Petersen LG, Juhler M (2015). Effect of postural changes on ICP in healthy and ill subjects. Acta Neurochir..

[45] Heldt T, Shim EB, Kamm RD, Mark RG (2002). Computational modeling of cardiovascular response to orthostatic stress. J. Appl. Physiol..

[46] Whittle RS, Diaz-Artiles A (2021). Modeling individual differences in cardiovascular response to gravitational stress using a sensitivity analysis. J. Appl. Physiol..

[47] Fois M (2022). Cardiovascular response to posture changes: multiscale modeling and in vivo validation during head-up tilt. Front. Physiol..

[48] Diaz-Artiles A, Heldt T, Young LR (2019). Computational model of cardiovascular response to centrifugation and lower body cycling exercise. J. Appl. Physiol..

[49] Gallo C, Ridolfi L, Scarsoglio S (2020). Cardiovascular deconditioning during long-term spaceflight through multiscale modeling. npj Microgravity.

[50] Mohammadyari P, Gadda G, Taibi A (2021). Modelling physiology of haemodynamic adaptation in short-term microgravity exposure and orthostatic stress on earth. Sci. Rep..

[51] Nelson ES (2017). The impact of ocular hemodynamics and intracranial pressure on intraocular pressure during acute gravitational changes. J. Appl. Physiol..

[52] Fois M, Ridolfi L, Scarsoglio S (2023). Arterial wave dynamics preservation upon orthostatic stress: a modelling perspective. R. Soc. Open Sci..

[53] Ursino M, Giannessi M (2010). A model of cerebrovascular reactivity including the circle of Willis and cortical anastomoses. Ann. Biomed. Eng..

[54] Anselmino M, Scarsoglio S, Saglietto A, Gaita F, Ridolfi L (2016). Transient cerebral hypoperfusion and hypertensive events during atrial fibrillation: a plausible mechanism for cognitive impairment. Sci. Rep..

[55] Scarsoglio S, Saglietto A, Anselmino M, Gaita F, Ridolfi L (2017). Alteration of cerebrovascular haemodynamic patterns due to atrial fibrillation: an in silico investigation. J. R. Soc. Interface.

[56] Saglietto A, Scarsoglio S, Ridolfi L, Gaita F, Anselmino M (2019). Higher ventricular rate during atrial fibrillation relates to increased cerebral hypoperfusions and hypertensive events. Sci. Rep..

[57] Scarsoglio S (2022). Cerebral hemodynamics during atrial fibrillation: computational fluid dynamics analysis of lenticulostriate arteries using 7t high-resolution magnetic resonance imaging. Phys. Fluids.

[58] Scarsoglio S, Fois M, Ridolfi L (2023). Increased hemodynamic pulsatility in the cerebral microcirculation during parabolic flight: a computational investigation. Acta Astronaut..

[59] Linder BJ, Trick GL, Wolf ML (1988). Altering body position affects intraocular pressure and visual function. Investig. Ophthalmol. Vis. Sci.

[60] Blomqvist, C. G. & Stone, H. L. *Cardiovascular Adjustments to Gravitational Stress. NASA*. Lyndon B. Johnson Space Center, Spacelab Life Sciences 1: Reprints of Background Life Sciences Publications (1991). URL: https://ntrs.nasa.gov/citations/19910016260.

[61] Montero D, Rauber S (2016). Brain perfusion and arterial blood flow velocity during prolonged body tilting. Aerosp. Med. Hum. Perform..

[62] Holmlund P (2018). Venous collapse regulates intracranial pressure in upright body positions. Am. J. Physiol. Regul. Integr. Comp. Physiol..

[63] Shi Y (2020). Small vessel disease is associated with altered cerebrovascular pulsatility but not resting cerebral blood flow. J. Cereb. Blood Flow Metab..

[64] Chung C-P, Lee H-Y, Lin P-C, Wang P-N (2017). Cerebral artery pulsatility is associated with cognitive impairment and predicts dementia in individuals with subjective memory decline or mild cognitive impairment. J. Alzheimer’s Dis..

[65] Nelson ES, Myers JG, Lewandowski BE, Ethier CR, Samuels BC (2020). Acute effects of posture on intraocular pressure. PLoS ONE.

[66] Peng M (2022). The ex vivo human translaminar autonomous system to study spaceflight associated neuro-ocular syndrome pathogenesis. npj Microgravity.

[67] Buckey JC, Lan M, Phillips SD, Archambault-Leger V, Fellows AM (2022). A theory for why the spaceflight-associated neuro-ocular syndrome develops. J. Appl. Physiol..

[68] Videbaek R, Norsk P (1997). Atrial distension in humans during microgravity induced by parabolic flights. J. Appl. Physiol..

[69] Buckey JC (2018). Microgravity-induced ocular changes are related to body weight. Am. J. Physiol. Regul. Integr. Comp. Physiol..

[70] Lan M (2021). Proposed mechanism for reduced jugular vein flow in microgravity. Physiol. Rep..

[71] Buckey J (1996). Central venous pressure in space. J. Appl. Physiol..

[72] Chiquet C (2003). Changes in intraocular pressure during prolonged (7-day) head-down tilt bedrest. J. Glaucoma.

[73] Harris KM, Petersen LG, Weber T (2020). Reviving lower body negative pressure as a countermeasure to prevent pathological vascular and ocular changes in microgravity. npj Microgravity.

[74] Petersen LG (2019). Lower body negative pressure to safely reduce intracranial pressure. Physiol. J..

[75] Hamilton DR (2012). Cardiac and vascular responses to thigh cuffs and respiratory maneuvers on crewmembers of the international space station. J. Appl. Physiol..

[76] Herault S (2000). Cardiac, arterial and venous adaptation to weightlessness during 6-month mir spaceflights with and without thigh cuffs (bracelets). Eur. J. Appl. Physiol..

[77] Morgan WH, Cunneen TS, Balaratnasingam C, Yu D-Y (2008). Wearing swimming goggles can elevate intraocular pressure. Br. J. Ophthalmol..

[78] Fois M, Ridolfi L, Scarsoglio S (2022). In silico study of the posture-dependent cardiovascular performance during parabolic flights. Acta Astronaut..

[79] Guala A, Camporeale C, Tosello F, Canuto C, Ridolfi L (2015). Modelling and subject-specific validation of the heart-arterial tree system. Ann. Biomed. Eng..

[80] Sit AJ, Nau CB, McLaren JW, Johnson DH, Hodge D (2008). Circadian variation of aqueous dynamics in young healthy adults. Investig. Ophthalmol. Vis. Sci..

[81] Williamson TH, Harris A (1994). Ocular blood flow measurement. Br. J. Ophthal..

[82] Saglietto A (2022). A computational analysis of atrial fibrillation effects on coronary perfusion across the different myocardial layers. Sci. Rep..

[83] Verhoeff K, Mitchell JR (2017). Cardiopulmonary physiology: why the heart and lungs are inextricably linked. Adv. Physiol. Educ..

[84] Du Bois D, Du Bois EF (1916). Clinical calorimetry: tenth paper a formula to estimate the approximate surface area if height and weight be known. Arch. Intern. Med..

