# Supplementary material for: Linking cerebral hemodynamics and ocular microgravity-induced alterations through an in silico-in vivo head-down tilt framework
Source: NPJ Microgravity. 2024 Feb 27;10:22. doi: 10.1038/s41526-024-00366-8 (PMC10899661; doi:10.1038/s41526-024-00366-8)
Supplement: Supplementary file 1 — Manuscrpt Suplementay Material [file 41526_2024_366_MOESM1_ESM.pdf]

Linking cerebral hemodynamics and ocular microgravity-induced alterations through an *in silico-in vivo* head-down tilt framework

SUPPLEMENTARY MATERIAL

Matteo Fois[a,*], Ana Diaz-Artiles[b,c], Syeda Yasmin Zaman[b], Luca Ridolfi[d,e], Stefania Scarsoglio[a,e]

[a]*Department of Mechanical and Aerospace Engineering, Politecnico di Torino, Corso Duca degli Abruzzi 24, Turin (10129), Italy*
[b]*Department of Aerospace Engineering, Texas A&M University, 3141 TAMU, College Station, TX (77843-3141)*
[c]*Department of Kinesiology and Sport Management, Texas A&M University, 2929 Research Pkwy, College Station, TX 77845*
[d]*Department of Environmental, Land and Infrastructure Engineering, Politecnico di Torino, Corso Duca degli Abruzzi 24, Turin (10129), Italy*
[e]*PolitoBioMed Lab, Politecnico di Torino, Corso Duca degli Abruzzi 24, Turin (10129), Italy*

Supplementary Methods

The global cardiovascular (CVS) model is composed of a 1D description of the arterial tree attached to 0D analogues of the systemic peripheral, venous, cardiopulmonary [1–3], coronary [4] and ocular-cerebrovascular circulations. The global model layout is illustrated in **Supplementary Figure 1**.

1D Arterial Tree. Blood motion through 1D arteries (arterial tree geometry and features given in **Supplementary Table 1**) is described by the 1D axisymmetric form of the Navier-Stokes equations for mass and momentum balance:

$$\frac{\partial A}{\partial t} + \frac{\partial Q}{\partial x} = 0\,, \tag{1}$$

$$\frac{\partial Q}{\partial t} + \frac{\partial}{\partial x}\left(\beta \frac{Q^2}{A}\right) + \frac{A}{\rho}\frac{\partial p}{\partial x} + N_4 \frac{Q}{A} + Ag\sin\gamma\sin\alpha = 0\,, \tag{2}$$

where $A(x,t)$ and $Q(x,t)$ are vessel cross-section area and blood flow rate, respectively, t is time and x the vessel axial coordinate. Blood is modeled as a Newtonian fluid with constant density $\rho = 1050$ Kg/m^3 and dynamic viscosity $\mu = 0.004$ Pa s. The Coriolis coefficient β and the viscous coefficient N_4 are computed assuming a flat-parabolic velocity profile over each vessel cross-section area.

Gravity is introduced in equations (1)-(2) through the term $g\sin\gamma\sin\alpha$, where g is gravity acceleration magnitude, γ is the vessel orientation with respect to the frontal transverse body axis and α is the vessel

*Corresponding author

inclination with respect to the horizontal reference (*i.e.*, the tilt angle).

The constitutive equation for blood (transmural) pressure $p(x,t)$ added to close the system (1)-(2) is a function of the local vessel cross-section area $A(x,t)$

$$P = B_1 + B_2 A + B_3 A^2 + B_4 A^3 - B_5 \frac{1}{\sqrt{A}} \frac{\partial Q}{\partial x}. \tag{3}$$

Here, coefficients B_i ($i = 1 \ldots 5$) are function of vessels' geometry and mechanical properties, through the local wave velocity at time $t = 0$ (c_0), and are defined as:

$$\begin{cases} B_1 = -\dfrac{1}{a_3^3}\left(a_5^3 + c_0^6 \rho^3 + 3c_0^4 \rho^2 a_5 + 3a_5^2 c_0^2 \rho\right), \\ B_2 = \dfrac{3\rho c_0^2}{A_0 a_3^3}\left(\rho^2 c_0^4 + 2\rho a_5 c_0^2 + a_5^2\right), \\ B_3 = -\dfrac{3\rho^2 c_0^4}{A_0^2 a_3^3}\left(a_5 + \rho c_0^2\right), \\ B_4 = \left(\dfrac{\rho c_0^2}{a_3 A_0}\right)^3, \\ B_5 = \dfrac{K_v h_w}{2\sqrt{A_0} r_0}, \end{cases}$$

where $a_3 = 1914$ N$^{2/3}$/m$^{4/3}$, $a_5 = -45348$ N/m^2, K_v is the effective viscosity of the wall, h_w is the wall thickness while A_0 and r_0 are the vessels cross-section area and radius at time $t = 0$, respectively. Local wave velocity is estimated as $c_0 = a_2/(2r_0)^{b_2}$ [5], where $a_2 = 13.3$ m$^{1.3}$/s and $b_2 = 0.3$. An additional partial-collapse hyperbolic model is introduced into eq. (3) for carotid and vertebral arteries (vessels numbered No. 6, 12, 13, 16, 17, 20) to cope with very low levels of transmural pressure [2].

Mass and total pressure conservation is imposed at inlet/outlet sections of arterial bifurcations:

$$\begin{cases} Q_{in} = Q_{out,1} + Q_{out,2}, \\ p_{in} + \dfrac{1}{2}\rho\left(\dfrac{Q}{A}\right)^2_{in} = p_{out,1} + \dfrac{1}{2}\rho\left(\dfrac{Q}{A}\right)^2_{out,1} = p_{out,2} + \dfrac{1}{2}\rho\left(\dfrac{Q}{A}\right)^2_{out,2}, \end{cases}$$

where subscripts in, $out,1$ and $out,2$ indicate the parent vessel and the corresponding two daughter vessels (three for the coronary bifurcation) of a bifurcation, respectively. A 0D model of the aortic valve (see valves model in section **0D Cardiopulmonary Circulation**) is coupled with the proximal aorta inlet section, whereas a 0D arteriolar compartment is plugged to each terminal 1D distal artery through a set

of lumped characteristic impedances $Z_c = \rho PWV_0/A_0$.

0D Peripheral Circulation and Venous Return. Equations governing blood motion in the (i, j)-th 0D compartment are

$$\frac{dV_{i,j}}{dt} = Q_{i,j-1} - Q_{i,j}\,, \tag{4}$$

$$\frac{dQ_{i,j}}{dt} = \begin{cases} \dfrac{p_{i,j} + \Delta p^h_{i,j} - R_{i,j}Q_{i,j} - p_{i,j+1}}{L_{i,j}}, & \text{if } j \in \{v, svc, ivc, avc\} \\ \dfrac{p_{i,j} - R_{i,j}Q_{i,j} - p_{i,j+1}}{L_{i,j}}, & \text{if } j \in \{art, cap, ven\} \end{cases}\,, \tag{5}$$

where subscript i denotes the considered body region (H, A, UA, LA, L), whereas j indicates the compartment (art, cap, ven, v, svc, ivc and avc). Intraluminal pressure of the (i, j)-th compartment is indicated with $p_{i,j}$, $Q_{i,j}$ is blood flow rate while $R_{i,j}$ and $L_{i,j}$ are compartmental hydraulic resistance and inertance, respectively. $V_{i,j}$ is the compartmental total blood volume, determined as

$$V_{i,j} = V^{un}_{i,j} + \big(p_{i,j} - p^{ext}_{i,j}\big)C_{i,j}\,, \tag{6}$$

where $C_{i,j}$ is compartmental compliance, whereas symbol $p^{ext}_{i,j}$ indicates either intrathoracic (ITP) or intracranial pressure (ICP). Where no external pressure is specified $p^{ext}_{i,j} = 0$ mmHg is assumed. The different constitutive law adopted to mimic non-linear effects of legs veins reads

$$V_{L,v} = V^{un}_{L,v} + \frac{2\Delta V_{max}}{\pi} \arctan\left(\frac{\pi C_{L,v}}{2\Delta V_{max}} p_{L,v}\right), \tag{7}$$

in which $\Delta V_{max} = 1200$ ml is the maximum distending volume of leg veins, $C_{L,v}$ is leg venous compliance. Values of 0D parameters are reported in **Supplementary Table 2**.

Hydrostatic pressure terms $\Delta p^h_{i,j}$ in eq. (5) are expressed according to Stevino's law:

$$\Delta p^h_{i,j} = \rho g \Delta h_{i,j} \sin\alpha\,, \tag{8}$$

where ρg is blood specific weight, $\Delta h_{i,j}$ is the hydrostatic height of the corresponding fluid column (apply only to v, svc, ivc and avc compartments, values reported in **Supplementary Table 3**), and α is the compartment orientation with respect to the horizontal reference (tilt angle).

0D Cardiopulmonary Circulation. Cardiac chambers are governed by the following constitutive equation

$$p_{ch} = E_{ch}\big(V_{ch} - V^{un}_{ch}\big) + ITP\,, \tag{9}$$

where cardiac transmural pressure $p_{ch} - ITP$ (ITP is intrathoracic pressure) is linked to the stressed volume $V_{ch} - V_{ch}^{un}$ (V_{ch}^{un} is the chamber's unstressed volume), whereas the elastance function E_{ch} is given by the relation:

$$E_{ch} = E_{ch,A} e_{ch} + E_{ch,B}\,, \tag{10}$$

in which parameters $E_{ch,A}$ and $E_{ch,B}$ denote chamber's elastance amplitude and baseline value. The normalized shape-elastance function $e_{ch}(t)$ of atria and ventricles are reported in [1].

Cardiac valves are described as non-ideal diodes accounting for several effects onto valve leaflets (i.e. on valve opening angle, θ_{va}) according to the following relationship:

$$\begin{cases} L_{va}\dfrac{dQ_{va}}{dt} + R_{va}Q_{va} + B_{va}|Q_{va}|Q_{va} = \dfrac{\big(1-\cos(\theta_{va})\big)^4}{\big(1-\cos(\theta_{va,max})\big)^4}\big(p_{va,u} - p_{va,d}\big)\,, & (11) \\ I_{va}\dfrac{d^2\theta_{va}}{dt^2} = K_{p,va}\big(p_{va,u} - p_{va,d}\big) + K_{q,va}Q_{va}\cos(\theta_{va}) - K_{f,va}\dfrac{d\theta_{va}}{dt} - K_{v,va}Q_{va}\sin(2\theta_{va})\,. & (12) \end{cases}$$

The various terms represent the effects of tissue friction ($K_{f,va} d\theta_{va}/dt$), pressure ($K_{p,va}(p_{va,u} - p_{va,d})$) and inertial ($K_{q,va}Q_{va}\cos(\theta_{va})$) forces, as the influence of downstream vortexes ($K_{v,va}Q_{va}\sin(2\theta_{va})$). B_{va} is Bernoulli's coefficient of the valve, R_{va} and L_{va} are valve resistance and inertance. Q_{va} is the transvalvular flow, $p_{va,u}$ and $p_{va,d}$ are upstream and downstream pressures governing the valve's leaflets (when $p_{va,u} - p_{va,d} \geq 0$ vortexes action is taken into account, otherwise it is neglected). Valve's coefficients K_p, K_q, K_f, K_v together with valve's model parameters are reported in [1].

Pulmonary arterial and venous compartments are governed by equations

$$\frac{dp_j}{dt} = \frac{Q_{j-1} - Q_j}{C_j} + \frac{dITP}{dt}\,, \tag{13}$$

$$Q_j = \frac{p_j - p_{j+1}}{R_j}\,, \tag{14}$$

where $j-1 = pu$, va (pulmonary valve) and $j+1 = pv$ (pulmonary veins) when $j = pa$ (pulmonary arteries), whereas $j-1 = pa$ and $j+1 = lv$ (left atrium) when $j = pv$. Values of pulmonary 0D parameters are reported in **Supplementary Table 2**

Intrathoracic Pressure. Intrathoracic pressure (*ITP*) applies to all cardiac chambers and pulmonary compartments. It varies with the body position (tilt angle α), according to the following relationship [2]

$$ITP = -4.014 + 1.127\frac{g}{g_0} + 0.895\left(\frac{g}{g_0}\right)^2 \sin\alpha - 4.508\frac{g}{g_0}\sin\alpha\,, \tag{15}$$

where g is the current gravity acceleration magnitude and $g_0 = 9.81$ m/s^2.

Multiscale Coronary Circulation. Specific 0D coronary microvascular districts are linked to each 1D large coronary artery outlet section to describe downstream vascular beds perfusing the myocardium. The coronary microvascular model is described in detail in [4].

Venous Valves. Venous valves are represented as non-linear hydraulic resistances and inertances within the arms and legs venous compartments, that is $R_{A,v}$, $R_{L,v}$, $L_{A,v}$ and $L_{L,v}$. Eq. (5) for the arms/legs venous compartments (subscript $A/L,v$) modifies as

$$\frac{dQ_{A/L,v}}{dt} = \frac{\xi_{A/L,v}(p_{A/L,v} + \Delta p^h_{A/L,v} - p_{svc/avc}) - R_{A/L,v}Q_{A/L,v} - B_{A/L,v}|Q_{A/L,v}|Q_{A/L,v}}{L_{A/L,v}}, \tag{16}$$

where $p_{A/L,v} + \Delta p^h_{A/L,v}$ and $p_{A/L,vc}$ are pressures immediately upstream and downstream the venous valve, while the function of the valve opening state $\xi_{A/L,v}$ is expressed as

$$\xi_{A/L,v} = \begin{cases} (1-\xi_{A/L,v})k_{vo}(p_{A/L,v} + \Delta p^h_{A/L,v} - p_{svc/avc}), & \text{if} \quad \Delta p_{A/L,v} \geq 0 \\ \xi_{A/L,v}k_{vc}(p_{A/L,v} + \Delta p^h_{A/L,v} - p_{svc/avc}), & \text{if} \quad \Delta p_{A/L,v} < 0 \end{cases}. \tag{17}$$

Parameters $k_{vo} = k_{vc} = 40$ 1/(mmHg s) are valve's opening and closing rate, respectively. $B_{A/L,v} = \rho/(2A^{eff}_{A/L,v})$ is the Bernoulli's coefficient, while $L_{A/L,v} = \rho(l^{eff}_{A/L,v}/A^{eff}_{A/L,v})$ and $R_{A/L,v} = 8\pi\mu/(A^{eff}_{A/L,v})^2$, with $l^{eff}_{A/L,v}$ assumed equal to the compartment radius, and the effective area $A^{eff}_{A/L,v}$ defined as

$$A^{eff}_{A/L,v} = \left(A^{eff,max}_{A/L,v} - A^{eff,min}_{A/L,v}\right)\xi_{A/L,v} + A^{eff,min}_{A/L,v}, \tag{18}$$

where $A^{eff,min}_{A/L,v} = 0$, $A^{eff,max}_{A/L,v} = A_{A/L,v}$, that is $V_{A/L,v}/L_{A/L}$ - ratio of the compartmental total blood volume and the characteristic length of the arms/legs venous compartments. Compartment radius can be determined from $A_{A/L,v}$ (assuming a circular compartment cross-section). Taking as reference the initial state of the system - for which $R_{A/L,v}(t=0) = R^0_{A/L,v}$ and $L_{A/L,v}(t=0) = L^0_{A/L,v}$, with venous valves completely open ($\xi_{A/L,v}(t=0) = 1$, $A^{eff}_{A/L,v}(t=0) = A^{eff,max}_{A/L,v}$) - the following expressions can be derived for the non-linear resistance $R_{A/L,v}$ and inertance $L_{A/L,v}$:

$$R_{A/L,v} = R^0_{A,L,v}\left(\frac{A^{eff,max}_{A/L,v}}{A^{eff}_{A/L,v}}\right)^2, \qquad L_{A/L,v} = L^0_{A/L,v}\frac{A^{eff,max}_{A/L,v}}{A^{eff}_{A/L,v}}. \tag{19}$$

Autonomic Control. The aortic-carotid sinus pressure $\bar{p}_{acs}$ sensed by the baroreflex control is obtained by averaging the pressure in the aortic arch ($p_{AA}(t)$) and the right/left carotid sinus pressure ($p_{cs,r}(t)$ and $p_{cs,l}(t)$) over the cardiac cycle:

$$\bar{p}_{acs} = \frac{1}{3RR}\int_{RR}\left(p_{AA}(t) + p_{cs,r}(t) + p_{cs,l}(t)\right)dt\,. \tag{20}$$

Cardiopulmonary receptors are located into the right atrium, sensing $\bar{p}_{cp}$ as the average right atrial pressure $\bar{p}_{ra}$:

$$\bar{p}_{cp} = \frac{1}{RR}\int_{RR} p_{ra}(t)\,dt\,. \tag{21}$$

Being $y_m(t)$ the generic parameter controlled by arterial baroreflex or cardiopulmonary reflex, its time evolution is governed by the differential equation:

$$\frac{dy_m}{dt} = \frac{1}{\tau_m}\Big(-y_m + \alpha_m n_s - \beta_m n_p + \gamma_m\Big)\,, \tag{22}$$

where symbols α_m, β_m, γ_m and τ_m are saturation and time delay parameters (values in [2]), whereas sympathetic (n_s) and parasympathetic (n_p) activity are

$$n_s = \frac{1}{1+\left(\dfrac{\bar{p}}{\bar{p}_{tg}}\right)^{\nu}} \quad , \quad n_p = \frac{1}{1+\left(\dfrac{\bar{p}}{\bar{p}_{tg}}\right)^{-\nu}}\,, \tag{23}$$

with $\nu = 7$, and $\bar{p}$ corresponding to either $\bar{p}_{acs}$ or $\bar{p}_{cp}$, while $\bar{p}_{tg}$ to $\bar{p}_{acs,tg}$ or $\bar{p}_{cp,tg}$.

Cerebrovascular Model. The lumped model of the cerebrovascular system is taken from [6] and encompasses 0D descriptions of the large cerebral arteries, of the distal pial circulation and intracerebral arterioles (distal arteries) and of the terminal capillary venous-circulation (see scheme in the Main Manuscript, Figure 5). All cerebrovascular model's details, equations and parameter values can be found in [7].

The cerebrovascular model is attached to the internal carotid and vertebral arteries (No. 6, 12, 16 and 20) of the global CVS model. The lumped characteristic impedance Z_c located at the end each 1D terminal artery to achieve the 1D-0D coupling has been virtually removed for arteries No. 6, 12, 16 and 20 of the global model. That is, Z_c has been imposed ~ 0 for internal carotid and vertebral arteries, as the corresponding 0D branching entering the lumped cerebrovascular model should still describe arterial tracts - not arteriolar as done for the rest of the global model. The pressure downstream the right and left internal carotid ($p_{ICA,r}$, $p_{ICA,l}$) and vertebral ($p_{VA,r}$, $p_{VA,l}$) arteries are used as input pressures for the

right and left internal carotid and basilar ($p_{BA} = 0.5\,(p_{VA,r} + p_{VA,l})$) arteries of the cerebrovascular model, respectively. Blood flow rates through the right and left internal carotid ($Q_{ICA,r}$, $Q_{ICA,l}$) and basilar (Q_{BA}) arteries are computed as

$$\begin{cases} Q_{ICA,r} = \dfrac{p_{ICA,r} - p_{MCA,r}}{R_{ICA,r}}\,, & (24) \\ Q_{ICA,l} = \dfrac{p_{ICA,l} - p_{MCA,l}}{R_{ICA,l}}\,, & (25) \\ Q_{BA} = \dfrac{p_{BA} - p_{BA,w}}{R_{BA}}\,, & (26) \end{cases}$$

where $p_{MCA,r}$, $p_{MCA,l}$ and $p_{BA,w}$ are right and left middle cerebral arteries and basilar (at the circle of Willis) pressures, determined from the pressure constitutive equation combined with the conservation of mass:

$$\begin{cases} C_{ICA,l}\left(\dfrac{dp_{MCA,l}}{dt} - \dfrac{dICP}{dt}\right) = Q_{ICA,l} + Q_{PCoA,l} - Q_{MCA,l} - Q_{ACA1,l}, & (27) \\ C_{ICA,r}\left(\dfrac{dp_{MCA,r}}{dt} - \dfrac{dICP}{dt}\right) = Q_{ICA,r} + Q_{PCoA,r} - Q_{MCA,r} - Q_{ACA1,r}, & (28) \\ C_{BA}\dfrac{dp_{BA,w}}{dt} = Q_{BA} - Q_{PCA1,l} - Q_{PCA1,r}, & (29) \end{cases}$$

where C are compartments compliance, Q are the blood flow rates entering and exiting the compartments, and ICP is intracranial pressure. $R_{ICA,r}$, $R_{ICA,r}$ and R_{BA} are right and left internal carotid and basilar arteries hydraulic resistances. The internal carotid and basilar arteries blood flow rates are used to complete the 1D-0D coupling as outflow condition for the corresponding 1D branches. 1D $Q_{ICA,r}$ and $Q_{ICA,l}$ are obtained as

$$\begin{cases} Q_{ICA,r,1D} = Q_{ICA,r} + Q_{eye,r}\,, & (30) \\ Q_{ICA,l,1D} = Q_{ICA,l} + Q_{eye,l}\,, & (31) \end{cases}$$

where $Q_{eye,r} = Q_{eye,l} = 0.5\,Q_{a,eye}$ (half the arterial eye input blood flow, see section **Ocular Compartment**). Right and left 1D vertebral arteries outflow is determined as $Q_{VA,r,1D} = Q_{VA,l,1D} = 0.5\,Q_{BA}$.

Blood flows through large cerebral and distal arteries are computed by Kirchhoff's law at nodes as

$$\begin{cases} Q_{ACA2,l} = Q_{ACA1,l} + Q_{ACoA}, \\ Q_{ACA2,r} = Q_{ACA1,r} - Q_{ACoA}, \\ Q_{PCA2,l} = -Q_{PCoA,l} + Q_{PCA1,l}, \\ Q_{PCA2,r} = -Q_{PCoA,r} + Q_{PCA1,r}, \end{cases}$$

or according to Ohm's law as

$$
\begin{cases}
Q_{MCA,l} = \dfrac{p_{MCA,l} - p_{dm,l}}{R_{MCA,l} + R_{dm,l}/2}, \\
Q_{MCA,r} = \dfrac{p_{MCA,r} - p_{dm,r}}{R_{MCA,r} + R_{dm,r}/2}, \\
Q_{ACA1,l} = \dfrac{p_{ICA,l} - p_{ACA,l}}{R_{ACA1,l}}, \\
Q_{ACA1,r} = \dfrac{p_{ICA,r} - p_{ACA,r}}{R_{ACA1,r}}, \\
Q_{PCA1,l} = \dfrac{p_{BA,w} - p_{PCA,l}}{R_{PCA1,l}}, \\
Q_{PCA1,r} = \dfrac{p_{BA,w} - p_{PCA,r}}{R_{PCA1,r}}, \\
Q_{ACA2,l} = \dfrac{p_{ACA,l} - p_{da,l}}{R_{ACA2,l} + R_{da,l}/2}, \\
Q_{ACA2,r} = \dfrac{p_{ACA,r} - p_{da,r}}{R_{ACA2,r} + R_{da,r}/2}, \\
Q_{ACoA} = \dfrac{p_{ACA,r} - p_{ACA,l}}{R_{ACoA}}, \\
Q_{PCA2,l} = \dfrac{p_{PCA,l} - p_{dp,l}}{R_{PCA2,l} + R_{dp,l}/2}, \\
Q_{PCA2,r} = \dfrac{p_{PCA,r} - p_{dp,r}}{R_{PCA2,r} + R_{dp,r}/2}, \\
Q_{PCoA,l} = \dfrac{p_{PCA,l} - p_{MCA,l}}{R_{PCoA,l}}, \\
Q_{PCoA,r} = \dfrac{p_{PCA,r} - p_{MCA,r}}{R_{PCoA,r}},
\end{cases}
$$

Distal compartmental blood volumes are obtained through mass conservation.

$$
\begin{cases}
\dfrac{dV_{dm,l}}{dt} = Q_{MCA,l} - Q_{dm,l} + Q_{cam,l} + Q_{cpm,l}, \\
\dfrac{dV_{dm,r}}{dt} = Q_{MCA,r} - Q_{dm,r} + Q_{cam,r} + Q_{cpm,r}, \\
\dfrac{dV_{da,l}}{dt} = Q_{ACA2,l} - Q_{da,l} - Q_{cam,l} + Q_{caa}, \\
\dfrac{dV_{da,r}}{dt} = Q_{ACA2,r} - Q_{da,r} - Q_{cam,r} - Q_{caa}, \\
\dfrac{dV_{dp,l}}{dt} = Q_{PCA2,l} - Q_{dp,l} - Q_{cpm,l} + Q_{cpp}, \\
\dfrac{dV_{dp,r}}{dt} = Q_{PCA2,r} - Q_{dp,r} - Q_{cpm,r} - Q_{cpp},
\end{cases}
$$

where flow rates Q are determined as

$$\begin{cases} Q_{dm,l} = \dfrac{p_{dm,l} - p_{ccap}}{R_{dm,l}/2}, \\ Q_{dm,r} = \dfrac{p_{dm,r} - p_{ccap}}{R_{dm,r}/2}, \\ Q_{da,l} = \dfrac{p_{da,l} - p_{ccap}}{R_{da,l}/2}, \\ Q_{da,r} = \dfrac{p_{da,r} - p_{ccap}}{R_{da,r}/2}, \\ Q_{dp,l} = \dfrac{p_{dp,l} - p_{ccap}}{R_{dp,l}/2}, \\ Q_{dp,r} = \dfrac{p_{dp,r} - p_{ccap}}{R_{dp,r}/2}, \\ Q_{cam,l} = \dfrac{p_{da,l} - p_{dm,l}}{R_{cam,l}}, \\ Q_{cam,r} = \dfrac{p_{da,r} - p_{dm,r}}{R_{cam,r}}, \\ Q_{cpm,l} = \dfrac{p_{dp,l} - p_{dm,l}}{R_{cpm,l}}, \\ Q_{cpm,r} = \dfrac{p_{dp,r} - p_{dm,r}}{R_{cpm,r}}, \\ Q_{caa} = \dfrac{p_{da,r} - p_{da,l}}{R_{caa}}, \\ Q_{cpp} = \dfrac{p_{dp,r} - p_{dp,l}}{R_{cpp}}, \end{cases}$$

Distal blood volumes are used to compute distal compartmental blood pressures according to pressure constitutive law:

$$\begin{cases} p_{dm,l} = \dfrac{V_{dm,l}}{C_{dm,l}} + ICP, \\ p_{dm,r} = \dfrac{V_{dm,r}}{C_{dm,r}} + ICP, \\ p_{da,l} = \dfrac{V_{da,l}}{C_{da,l}} + ICP, \\ p_{da,r} = \dfrac{V_{da,r}}{C_{da,r}} + ICP, \\ p_{dp,l} = \dfrac{V_{dp,l}}{C_{dp,l}} + ICP, \\ p_{dp,r} = \dfrac{V_{dp,r}}{C_{dp,r}} + ICP, \end{cases}$$

where p and V are the compartment blood pressure and volume.

The downstream capillary-venous circulation is connected to the superior vena cava of the global CVS model. The dural venous sinus pressure (p_{dvs}) applied to the outflow branch of the cerebrovascular model is determined as

$$p_{dvs} = p_{svc} - \rho g \left(\frac{L_H}{2} + \frac{L_{svc}}{2} \right) \sin \alpha \,, \tag{32}$$

where p_{svc} is superior vena cava pressure, ρg is blood specific weight, α is the tilt angle and L_H and L_{svc} are the head and superior vena cava compartment anatomical extensions (**Supplementary Table 3**), respectively. p_{dvs} is used to compute the venous sinus blood flow (Q_{vs}) and the cerebrospinal fluid outflow rate (Q_o) through Ohm's law as:

$$\begin{cases} Q_{cv} = \dfrac{p_{ccap} - p_{cv}}{R_{pv}}, & (33) \\ Q_{dvs} = \dfrac{p_{cv} - p_{dvs}}{R_{dvs}}, & (34) \\ Q_f = \begin{cases} \dfrac{p_{ccap} - ICP}{R_f}, \text{ if } p_{ccap} \geq ICP, \\ 0, \text{ if } p_{ccap} < ICP, \end{cases} & (35) \\ Q_o = \begin{cases} \dfrac{ICP - p_{dvs}}{R_o}, \text{ if } ICP \geq p_{dvs}, \\ 0, \text{ if } ICP < p_{dvs}, \end{cases} & (36) \\ CBF = Q_{dvs} + Q_o, & (37) \end{cases}$$

where CBF is cerebral blood flow, the overall blood flow drained from the brain. The cerebrospinal fluid rate of formation (Q_f) depends instead on the cerebral capillary pressure p_{ccap}, whereas the cerebral venous pressure is computed through the pressure constitutive law combined with mass conservation:

$$C_{cv} \left(\frac{dp_{cv}}{dt} - \frac{dICP}{dt} \right) = Q_{cv} - Q_{dvs}, \tag{38}$$

with

$$\begin{cases} C_{ic} = \dfrac{1}{k_E ICP}, \\ C_{cv} = \dfrac{1}{k_{ven}(p_{cv} - ICP - p_{v1})}, \\ R_{dvs} = \begin{cases} \dfrac{p_{cv} - p_{dvs}}{p_{cv} - ICP} R_{dvs1}, \text{ if } p_{dvs} < ICP, \\ R_{dvs1}, \text{ if } p_{dvs} \geq ICP, \end{cases} \end{cases}$$

and

$$Q_{dm,l} + Q_{da,l} + Q_{dp,l} + Q_{dm,r} + Q_{da,r} + Q_{dp,r} = Q_f + Q_{cv},$$

Intracranial pressure (ICP) is given by the pressure constitutive law combined with mass conservation, taking into account the action of gravity through the cerebrospinal fluid hydrostatic pressure (Stevino's law), so that

$$\begin{aligned} C_{ic}\frac{dICP}{dt} &= Q_{MCA,left} - Q_{dm,l} + Q_{PCA2,l} - Q_{dp,l} + Q_{ACA2,l} - Q_{da,l} + \\ &+ Q_{MCA,r} - Q_{dm,r} + Q_{PCA2,r} - Q_{dp,r} + Q_{ACA2,r} - Q_{da,r} + \\ &+ Q_{cv} - Q_{dvs} + Q_f - Q_o - C_{ic}\frac{dICP^h}{dt}, \end{aligned} \tag{39}$$

where

$$ICP^h = \rho g \left(\frac{L_H}{2} + \frac{L_{svc}}{2}\right)\sin\alpha, \tag{40}$$

with C_{ic} the intracranial non-linear compliance and Q terms the blood flow rates entering and exiting the compartments communicating with the intracranial cavity.

Cerebral (distal) arteriolar resistances and compliances are controlled by cerebral autoregulation and CO_2 reactivity . To each of the six distal regions, the following equations apply:

$$\begin{cases} \tau_{aut}\frac{dx_{aut,i,j}}{dt} = -x_{aut,i,j} + G_{aut}\left(\frac{Q_{di,j} - Q_{ndi,j}}{Q_{ndi,j}}\right), \text{ i=m,a,p; j=l,r,} & (41) \\ \tau_{CO_2}\frac{dx_{CO_2,i,j}}{dt} = -x_{CO_2,i,j} + G_{CO_2}A_{CO_2,i,j}\log_{10}\left(\frac{p_{aCO_2}}{p_{aCO_2n}}\right), \text{ i=m,a,p; j=l,r,} & (42) \end{cases}$$

where the subscript n denotes the basal values and

$$A_{CO_2,i,j} = \frac{1}{1+\exp\{[-k_{CO_2}(Q_{di,j} - Q_{ndi,j})/Q_{ndi,j}] - b_{CO_2}\}}, \text{ i=m,a,p; j=l,r.} \tag{43}$$

Distal compliances and resistances are expressed as

$$\begin{cases} C_{di,j} = \frac{C_{d0i,j}\left[(1-\Delta C_{di,j}/2) + (1+\Delta C_{di,j}/2)e^{\frac{x_{CO_2,i,j} - x_{aut,i,j}}{k_{C_{di,j}}}}\right]}{1+e^{\frac{x_{CO_2,i,j} - x_{aut,i,j}}{k_{C_{di,j}}}}}, & (44) \\ R_{di,j} = \frac{k_{R_{di,j}}C^2_{d0i,j}}{V^2_{di,j}}, \text{ i=m,a,p; j=l,r,} & (45) \end{cases}$$

where

$$\Delta C_{di,j} = \begin{cases} 2s_1, \text{ if } x_{CO_2,i,j} < x_{aut,i,j}, \\ 2s_2, \text{ if } x_{CO_2,i,j} \geq x_{aut,i,j}, \end{cases} \quad \text{i=m,a,p; j=left,right}$$

$$k_{C_{di,j}} = \begin{cases} \dfrac{C_{d0i,j}s_1}{2}, \text{ if } x_{CO_2,i,j} < x_{aut,i,j}, \\ \dfrac{C_{d0i,j}s_2}{2}, \text{ if } x_{CO_2,i,j} \geq x_{aut,i,j}, \end{cases} \quad \text{i=m,a,p; j=l,r}$$

Parameters and initial values of terms appearing in the cerebrovascular model equations can be found in **Tables 4-7** and in [7].

Ocular Compartment. The lumped model of the ocular compartment is adopted from [8, 9]. The ocular model is multi-compartment and regulates the behavior of the intraocular pressure (IOP) and of the ocular globe volume (V_g) through the following governing equations:

$$\frac{dIOP}{dt} = \frac{1}{C_g}\left(C_{rg}\frac{dICP}{dt} + C_{ag}\frac{dp_{a,eye}}{dt} + C_{vg}\frac{dp_{v,eye}}{dt} + Q_{aq,in} + C_{tm}EVP - Q_{uv} - C_{tm}IOP\right), \quad (46)$$

$$\frac{dV_g}{dt} = C_{ag}\frac{d}{dt}\big(p_{a,eye} - IOP\big) + C_{vg}\frac{d}{dt}\big(p_{v,eye} - IOP\big) + Q_{aq,in} + C_{tm}(EVP - IOP) - Q_{uv}, \quad (47)$$

where the globe compliance $C_g = V_g(C_1/IOP + C_2)$ (constants $C_1 = 4.87e-3, C_2 = 3.90e-5$ 1/mmHg), the retrobulbar subarachnoid space-to-globe compliance $C_{rg} = 1.1e-9$ l/mmHg, the arterial blood-to-globe compliance $C_{ag} = 0.3V_g(C_1/IOP + C_2 - 1/(k_g IOP))$ and venous blood-to-globe compliance $C_{vg} = 0.7V_g(C_1/IOP + C_2 - 1/(k_g IOP))$ (with $k_g = 312$ the non-dimensional globe stiffness), the aqueous humor formation rate $Q_{aq,in} = 0.048e-6$ l/s, the aqueous outflow facility $C_{tm} = 0.0035e-6$ l/(s mmHg) (as long as $EVP \leq IOP$, 0 otherwise), the uveoscleral outflow rate $Q_{uv} = 0.0067e-6$ l/s (parameters adjusted according to physiological values [10]). $p_{a,eye}$ and $p_{v,eye}$ are arterial and venous pressure at the level of the eye and episcleral venous pressure, respectively, taken as

$$p_{a,eye} = 0.5(p_{ICA,r} + p_{ICA,l}) - L_{f-b}\rho g \cos\alpha, \quad (48)$$

$$p_{v,eye} = \max\big(CVP, EVP\big), \quad (49)$$

$$EVP = p_{svc} - \rho g\left(\frac{L_H}{2} + \frac{L_{svc}}{2}\right)\sin\alpha - L_{f-b}\rho g \cos\alpha, \quad (50)$$

where $L_{f-b} = 0.03$ m is the perpendicular distance between the globe and the mid-coronal plane.

To integrate the ocular model with the global CVS, the arterial eye input and venous eye output flow rates shall be determined. These are obtained from the mass conservation and the pressure constitutive law applied to the ocular arterial and venous blood compartments as

$$Q_{a,eye} = C_{ag}\frac{d}{dt}\left(dp_{a,eye} - IOP\right) + Q_{eye}\,, \tag{51}$$

$$Q_{v,eye} = Q_{eye} - C_{vg}\frac{d}{dt}\left(dp_{v,eye} - IOP\right)\,, \tag{52}$$

with the eye blood flow rate $Q_{eye} = (p_{a,eye} - p_{v,eye})/R_{eye}$, and the eye resistance $R_{eye} = 4676$ mmHg s/ml such that mean $Q_{eye} \simeq 1$ ml/min [11].

Numerical Simulation. 1D governing equations are discretized and integrated numerically according to a Discontinuous Galerkin Finite Elements approach. The solution is advanced in time employing a 2-step Runge-Kutta explicit scheme with constant time step [1]. Ordinary differential equations governing 0D compartments are advanced in time via the same 2-step Runge-Kutta explicit scheme.

Supplementary Figures

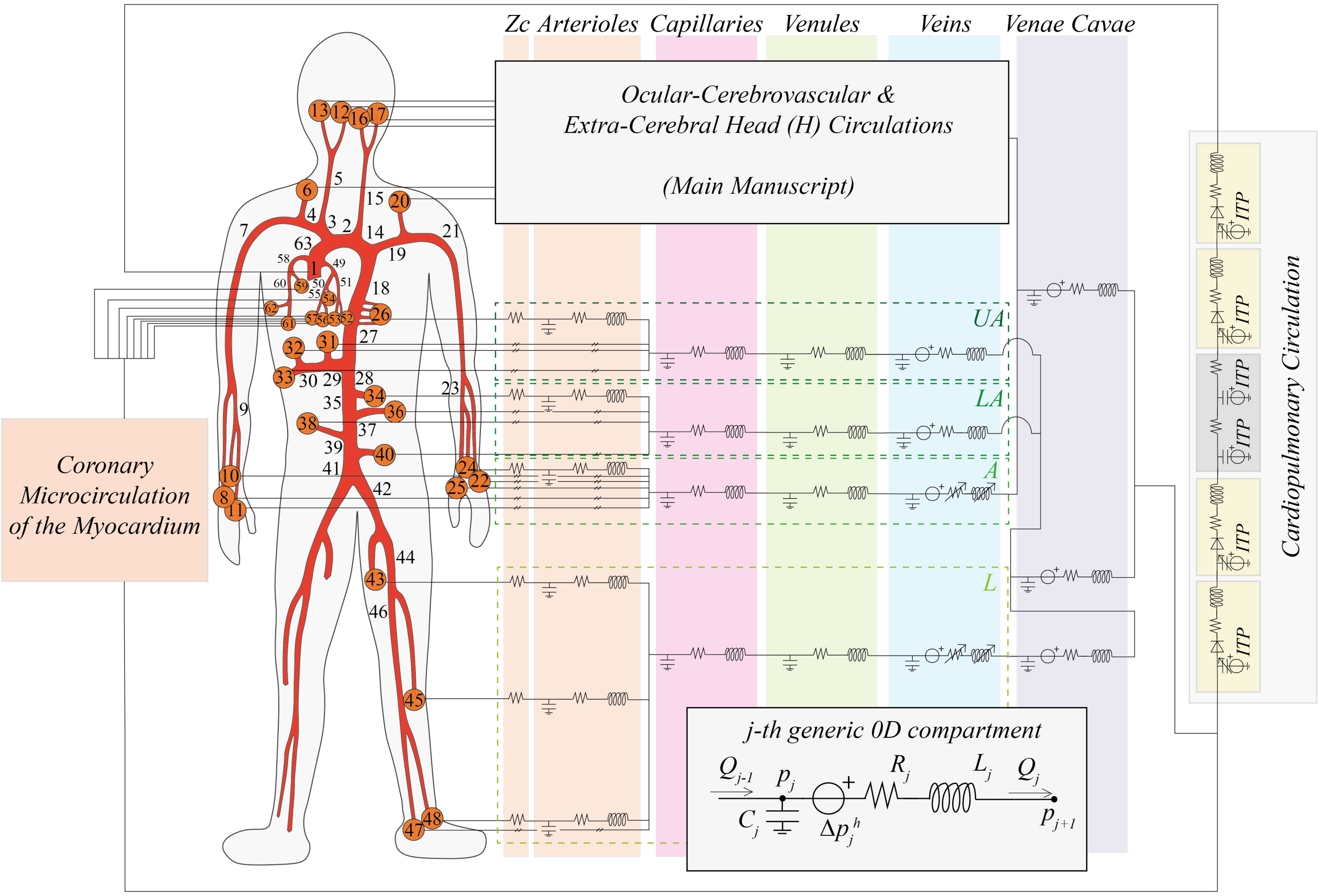

Supplementary Figure 1: **Global CVS model.** Illustration of the global multiscale cardiovascular model. The 1D arterial tree is sketched in red, black numbers refer to 1D main arterial vessels (**Supplementary Table 1**), numbers in orange circles denote 1D terminal arteries linked to the downstream 0D compartments, grouped into regions from head to legs (H, UA, LA, A, L). Vertical rectangles (orange, pink, green and blue, from left to right) divide the 0D peripheral circulation into arteriolar, capillary, venular, venous and venae cavae compartments. Z_c are arteriolar characteristic impedances, while R_j, C_j and L_j parameters indicate vessels' lumped resistance, compliance and inertance, respectively, of the j-th generic compartment. Q_j and p_j are generic compartmental blood flow rate and pressure (Q_{j-1} and p_{j+1} refer to the previous and following compartment), Δp_j^h is the gravitational hydrostatic contribution. The cardiopulmonary circulation is represented on the right (ITP is intrathoracic pressure).

Supplementary Tables

Supplementary Table 1: **1D geometry.** Geometric features of the 1D arterial tree (l: vessel length, D_{in} : vessel inlet diameter, D_{out}: vessel outlet diameter, h_{wall}: vessel wall thickness, γ: vessel orientation with respect to the frontal transverse body axis).

No.	Artery	l [mm]	D_{in} [mm]	D_{out} [mm]	h_{wall} [mm]	γ [°]
1	Ascending Aorta I	10	29.40	29.30	1.63	-90
2	Aortic Arch I	20	24.10	24	1.32	0
3	Brachiocephalic Artery	34	19.40	18	0.86	-45
4/19	R/L Subclavian Artery I	34	12.90/11	9/8.5	0.67	-30/-45
5/15	R/L Common Carotid Artery	94/119	15.10/12.40	7/6	0.63	-90
6/20	R/L Vertebral Artery	149/148	4.10/3.80	2.80	0.45	-90
7/21	R/L Subclavian II, Axillary & Brachial Arteries	422	8.90/8.40	4.70	0.66	+90
8/22	R/L Radial Artery	235	3.70/3.30	3.10/2.80	0.43	+90
9/23	R/L Ulnar Artery I	67	3.70/4	3.40/4	0.49	+90
10/24	R/L Interosseous Artery	79	2.10/1.80	1.80	0.28	+90
11/25	R/L Ulnar Artery II	171	3.20/4.10	2.80/3.70	0.49	+90
12/16	R/L Internal Carotid Artery	178	5.70/5	4.30/4.30	0.39	-90
13/17	R/L External Carotid Artery	41	5/4.50	4.50/4.30	0.42	-90
14	Aortic Arch II	39	22	20.80	1.27	0
18	Thoracic Aorta I	52	20	18.90	1.20	+90
26	Intercostal Arteries	80	12.60	9.50	1.20	0
27	Thoracic Aorta II	104	16.50	12.90	1.16	+90
28	Abdominal Aorta I	53	12.20	12.20	1.08	+90
29	Celiac Artery I	20	7.80	6.90	0.64	0
30	Celiac Artery II	25	5.20	4.90	0.64	0
31	Hepatic Artery	66	5.40	4.40	0.49	0
32	Gastric Artery	71	3.20	3	0.45	-90
33	Splenic Artery	63	4.20	3.90	0.54	0
34	Superior Mesenteric Artery	59	7.90	7.10	0.69	+90
35	Abdominal Aorta II	20	11.50	11.30	0.8	+90
36/38	R/L Renal Artery	32	4.94	4.94/5.2	0.53	0
37	Abdominal Aorta III	20	11.20	11.20	0.8	+90
39	Abdominal Aorta IV	106	11	11	0.8	+90
40	Inferior Mesenteric Artery	50	4.70	3.20	0.43	+90
41	Abdominal Aorta V	20	10.80	10.40	0.8	+90
42	Common Iliac Artery	59	7.90	7	0.76	+45
43	Inner Iliac Artery	50	4	4	0.4	+45
44	External Iliac Artery	144	6.40	6.10	0.55	+90
45	Deep Femoral Artery	126	4	3.70	0.49	+90
46	Femoral Artery	443	5.20	3.80	0.5	+90
47	Anterior Tibial Artery	343	2.60	2.30	0.39	+90
48	Posterior Tibial Artery	321	3.10	2.80	0.45	+90
49	Left Main Coronary Artery	12	4.50	4.50	0.4	0
50	Left Anterior Descending Coronary Artery I	18	3.74	3.74	0.4	+60
51	Circumflex Coronary Artery I	32	3.60	3.60	0.4	+60
52	Marginal Coronary Artery	73	2.52	2.52	0.3	+90
53	Circumflex Coronary Artery II	43	3	3	0.2	+90
54	Diagonal Coronary Artery	65	2.40	2.40	0.3	+90
55	Left Anterior Descending Coronary Artery II	22	3.29	3.29	0.3	+90
56	Septal Coronary Artery II	45	2.39	2.39	0.2	0
57	Left Anterior Descending Coronary Artery III	100	2.72	2.72	0.3	+90
58	Right Coronary Artery I	50	4.05	4.05	0.4	+60
59	Acute Marginal Coronary Artery I	34	2.52	2.52	0.3	0
60	Right Coronary Artery II	48	3.55	3.55	0.4	+90
61	Acute Marginal Coronary Artery II	23	2.11	2.11	0.2	+60
62	Right Coronary Artery III (Posterior Descending Artery)	89	3.21	3.21	0.3	+60
63	Ascending Aorta II	30	29.3	28.8	1.63	-90

Supplementary Table 2: **0D parameters values**. *R*, *C*, *L*, V^{un} and V are lumped compartmental hydraulic resistance, compliance, inertance, unstressed volume and total blood volume, respectively. *H*, *A*, *UA*, *LA* and *L* refer to the head, arms, upper and lower abdomen and legs body regions. *svc*, *ivc* and *avc* are superior, inferior and abdominal veane cavae compartments, while *pa* and *pv* denote pulmonary arterial and venous compartments, respectively.

Compartment	*R* [mmHg s/ml]	*C* [mmHg/ml]	*L* [mmHg s/ml]	V^{un} [ml]	*V* [ml]
Capillary					
H	5.0048	0.0029	0.00150	2.50	2.58
A	1.8876	0.0058	0.00065	46.71	46.88
UA	1.0611	0.0121	0.00051	111.64	112.00
LA	0.5598	0.0226	0.00039	71.32	72.00
L	0.9550	0.0140	0.00037	56.86	57.28
Venule					
H	1.6038	0.0853	0.00260	8.67	9.69
A	0.6043	0.1780	0.00120	173.66	175.80
UA	0.3397	0.4470	0.00093	414.64	420.00
LA	0.1784	0.6760	0.00096	261.89	270.00
L	0.3061	0.5500	0.00061	208.20	214.80
Vein					
H	0.6653	0.91	0.00290	20.03	20.03
A	0.2200	3.71	0.00130	333.64	363.32
UA	0.0653	49.50	0.00100	515.20	868.00
LA	0.0455	23.80	0.00078	367.60	558.00
L	0.1422	18.00	0.00067	299.92	443.92
Vena Cava					
svc	0.00055	5.0	0.00005	30	60
ivc	0.00055	7.5	0.00005	55	100
avc	0.00055	7.5	0.00005	15	60
Pulmonary					
pa	0.08	3.8	-	44.3	89.9
pv	0.005	20.5	-	232.8	396.8

Supplementary Table 3: **Venous/venae cavae anatomical lengths.** Anatomical length of head (*H*), arms (*A*), lower/upper abdomen (*LA*, *UA*), legs (*L*) venous and venae cavae (*svc*, *ivc*, *avc*) compartments.

Compartment	Length [m]
H	0.20
A	0.55
UA	0
LA	0
L	1.00
svc	0.07
ivc	0.15
avc	0.07

Supplementary Table 4: **Large Arteries.** Parameters values (R: resistances, C: compliances) of large cerebral arteries.

Parameter	Value
$R_{ICA,l}$	0.5689 mmHg s/ml
$R_{ICA,r}$	0.5689 mmHg s/ml
R_{BA}	0.4501 mmHg s/ml
$R_{MCA,l}$	1.4419 mmHg s/ml
$R_{MCA,r}$	1.4419 mmHg s/ml
$R_{PCA1,l}$	0.7640 mmHg s/ml
$R_{PCA1,r}$	0.7640 mmHg s/ml
$R_{ACA1,l}$	3.7912 mmHg s/ml
$R_{ACA1,r}$	3.7912 mmHg s/ml
$R_{PCA2,l}$	3.6063 mmHg s/ml
$R_{PCA2,r}$	3.6063 mmHg s/ml
$R_{ACA2,l}$	1.6227 mmHg s/ml
$R_{ACA2,r}$	1.6227 mmHg s/ml
$R_{PCoA,l}$	90.9786 mmHg s/ml
$R_{PCoA,r}$	90.9786 mmHg s/ml
R_{ACoA}	14.9228 mmHg s/ml
$C_{ICA,l}$	0.0034 ml/mmHg
$C_{ICA,r}$	0.0034 ml/mmHg
C_{BA}	0.0017 ml/mmHg

Supplementary Table 5: **Distal Arteries.** Parameters values (R: resistances) of distal cerebral arteries.

Parameter	Value
$R_{cam,l}$	105 mmHg s/ml
$R_{cam,r}$	105 mmHg s/ml
$R_{cpm,l}$	120 mmHg s/ml
$R_{cpm,r}$	120 mmHg s/ml
R_{caa}	22 mmHg s/ml
R_{cpp}	75 mmHg s/ml

Supplementary Table 6: **Capillary-venous.** Parameters values (R: resistances, k: constants, p: pressures) of cerebral capillary-venous circulation.

Parameter	Value
R_f	$2.3 \cdot 10^3$ mmHg s/ml
R_o	526.3 mmHg s/ml
R_{cv}	0.880 mmHg s/ml
R_{dvs1}	0.366 mmHg s/ml
k_E	0.077 ml^{-1}
k_{ven}	0.155 ml^{-1}
p_{v1}	-2.5 mmHg

Supplementary Table 7: **Cerebral control mechanisms.** Parameters values (τ: time-delay parameters, G: gains, k, s and b: constants, Q: flow rates, C: compliances, p: pressures) of cerebral autoregulation and CO_2 reactivity control mechanisms.

Parameter	Value
τ_{aut}	20 s
G_{aut}	0.9
τ_{CO_2}	40 s
G_{CO_2}	4.0
k_{CO_2}	15
b_{CO_2}	0.5
P_{aCO_2n}	40 mmHg
P_{aCO_2}	40 mmHg
s_1	7
s_2	0.4
$Q_{ndm,left}$	3.75 ml/s
$Q_{ndm,right}$	3.75 ml/s
$Q_{nda,left}$	1 ml/s
$Q_{nda,right}$	1 ml/s
$Q_{ndp,left}$	1.5 ml/s
$Q_{ndp,right}$	1.5 ml/s
$k_{R_{dm,left}}$	$3.4278 \cdot 10^{4}$ mmHg^{-3} s/ml
$k_{R_{dm,right}}$	$3.4278 \cdot 10^{4}$ mmHg^{-3} s/ml
$k_{R_{da,left}}$	$1.2848 \cdot 10^{5}$ mmHg^{-3} s/ml
$k_{R_{da,right}}$	$1.2848 \cdot 10^{5}$ mmHg^{-3} s/ml
$k_{R_{dp,left}}$	$0.8622 \cdot 10^{5}$ mmHg^{-3} s/ml
$k_{R_{dp,right}}$	$0.8622 \cdot 10^{5}$ mmHg^{-3} s/ml
$C_{d0m,left}$	0.06 ml/mmHg
$C_{d0m,right}$	0.06 ml/mmHg
$C_{d0a,left}$	0.016 ml/mmHg
$C_{d0a,right}$	0.016 ml/mmHg
$C_{d0p,left}$	0.024 ml/mmHg
$C_{d0p,right}$	0.024 ml/mmHg

Supplementary Table 8: **Tests of significance.** P-values resulting from statistical test of significance (two-tailed Wilcoxon paired test, $n = 6$). BL denotes the baseline seated posture, MAP indicates mean arterial pressure (taken at the finger and corrected at brachial level), HR denotes the heart rate, CO indicates cardiac output, SV indicates stroke volume, and IOP indicates the intraocular pressure.

Parameter	BL vs. 80° HUT pre-tilt	80° HUT pre-tilt vs. 6° HDT	6° HDT vs. 80° HUT post-tilt	80° HUT post-tilt vs. 80° HUT pre-tilt
MAP	0.4688	0.0938	0.0312	0.5000
HR	0.0625	0.0312	0.0312	0.1875
CO	0.0625	0.0312	0.0312	0.1562
SV	0.0312	0.0312	0.0312	0.1562
IOP	0.0938	0.0312	0.0312	0.3125

Supplementary Table 9: **Normalized peak pressures and flow rates during overshoots.** Peak cerebrovascular pressure and flow rates overshoots during a HDT maneuver normalized by the post-tilt steady-state value at 6° HDT. p_{peak} and Q_{peak} indicate peak pressure and flow rate during overshoots, while $p_{6°HDT}$ and $Q_{6°HDT}$ indicate post-tilt steady-state pressure and flow rate values at 6° HDT, respectively.

Parameter	$p_{peak}/p_{6°HDT}$	Parameter	$Q_{peak}/Q_{6°HDT}$
$p_{ICA,l}$	1.09	$Q_{ICA,l}$	1.22
$p_{MCA,l}$	1.09	$Q_{MCA,l}$	1.21
$p_{dm,l}$	1.05	$Q_{dm,l}$	1.26
p_{ccap}	1.02	Q_{cv}	1.22
p_{cv}	1.02	CBF	1.22

Supplementary Table 8 reports the results of statistical tests of significance – for each measured parameter – between the different phases of the experiment. For all parameters, pre- and post-tilt 80° HUT positions are non-significantly different from each other ($p > 0.05$). Conversely, most parameters show statistically significant differences between pre- and post-tilt 80° HUT and 6° HDT positions ($p < 0.05$; with only *MAP* $p < 0.10$ between pre-tilt 80° HUT and 6° HDT).

To better quantify the peak pressure and flow rate overshoots observed during the transient dynamics of HDT, **Supplementary Table 9** shows the p_{peak}-to-$p_{6°HDT}$ and Q_{peak}-to-$Q_{6°HDT}$ ratios for all considered sites (left *ICA*, *MCA* and *dm*, capillary, and venous). Results show that, in general, normalized flow rate overshoots are twice as large as the normalized pressure overshoots (1.21 - 1.26 for flow rates vs. 1.02 - 1.09 for pressures) likely because of the prompt intervention of cerebral autoregulation limiting flow rate growth at post-tilt 6° HDT.

References

[1] M. Fois, S. V. Maule, M. Giudici, M. Valente, L. Ridolfi, S. Scarsoglio, Cardiovascular response to posture changes: Multiscale modeling and in vivo validation during head-up tilt, Frontiers in Physiology 13 (826989) (2022). doi:10.3389/fphys.2022.826989.

[2] M. Fois, L. Ridolfi, S. Scarsoglio, In silico study of the posture-dependent cardiovascular performance during parabolic flights, Acta Astronautica 200 (2022) 435–447. doi:10.1016/j.actaastro.2022.08.018.

[3] M. Fois, L. Ridolfi, S. Scarsoglio, Arterial wave dynamics preservation upon orthostatic stress: a

modelling perspective, Royal Society Open Science 10 (3) (2023) 221257. doi:10.1098/rsos.221257.

[4] A. Saglietto, M. Fois, L. Ridolfi, G. M. De Ferrari, M. Anselmino, S. Scarsoglio, A computational analysis of atrial fibrillation effects on coronary perfusion across the different myocardial layers, Scientific reports 12 (841) (2022). doi:10.1038/s41598-022-04897-6.

[5] A. Guala, C. Camporeale, F. Tosello, C. Canuto, L. Ridolfi, Modelling and subject-specific validation of the heart-arterial tree system, Annals of biomedical engineering 43 (1) (2015) 222–237. doi:10.1007/s10439-014-1163-9.

[6] M. Ursino, M. Giannessi, A model of cerebrovascular reactivity including the circle of willis and cortical anastomoses, Annals of biomedical engineering 38 (2010) 955–974. doi:10.1007/s10439-010-9923-7.

[7] S. Scarsoglio, M. Fois, L. Ridolfi, Increased hemodynamic pulsatility in the cerebral microcirculation during parabolic flight: a computational investigation, (under review).

[8] E. S. Nelson, L. Mulugeta, A. Feola, J. Raykin, J. G. Myers, B. C. Samuels, C. R. Ethier, The impact of ocular hemodynamics and intracranial pressure on intraocular pressure during acute gravitational changes, Journal of Applied Physiology 123 (2) (2017) 352–363. doi:10.1152/japplphysiol.00102.2017.

[9] L. G. Petersen, R. S. Whittle, J. H. Lee, J. Sieker, J. Carlson, C. Finke, C. M. Shelton, J. C. Petersen, A. Diaz-Artiles, Gravitational effects on intraocular pressure and ocular perfusion pressure, Journal of Applied Physiology 132 (1) (2022) 24–35. doi:10.1152/japplphysiol.00546.2021.

[10] A. J. Sit, C. B. Nau, J. W. McLaren, D. H. Johnson, D. Hodge, Circadian variation of aqueous dynamics in young healthy adults, Investigative ophthalmology & visual science 49 (4) (2008) 1473–1479. doi:10.1167/iovs.07-1139.

[11] T. H. Williamson, A. Harris, Ocular blood flow measurement., The British Journal of Ophthalmology 78 (12) (1994) 939. doi:10.1136/bjo.78.12.939.
